# Age, Sex, and Waist-to-Height Ratio Approach the Discrimination of Fifty-Four Model Inputs for Prevalent Hypertension: An Explainable Machine Learning Analysis of the Chilean National Health Survey

**DOI:** 10.3390/diagnostics16172843

**Published:** 2026-09-04

**Authors:** Rodrigo Yáñez-Sepúlveda, Boryi A. Becerra-Patiño, Felipe Montalva-Valenzuela, Rodrigo Olivares, Alejandra Uribe-Díaz, Eduardo Guzmán-Muñoz, Yeny Concha-Cisternas, Daniel Rojas-Valverde, José Francisco Tornero-Aguilera, Vicente Javier Clemente-Suárez, José Francisco López-Gil

**Affiliations:** 1 Faculty of Education and Humanities, Universidad Andrés Bello, Vina del Mar 2520000, Chile; rodrigo.yanez.s@unab.cl; 2 Department of Sport Sciences, Faculty of Sport and Health Sciences, Fit Generation Research Institute, AD500 Andorra la Vella, Andorra; jtornero@fitgeneration.es (J.F.T.-A.); vctxente@yahoo.es (V.J.C.-S.); 3 Facultad de Educación Física, Universidad Pedagógica Nacional, Bogota 480100, Colombia; babecerrap@pedagogica.edu.co; 4 Escuela de Entrenador en Actividad Física y Deporte, Facultad de Ciencias Humanas, Universidad Bernardo O’Higgins, Santiago 8370040, Chile; felipe.montalva@ubo.cl; 5 Escuela de Ingeniería Informática, Universidad de Valparaíso, Valparaiso 2340000, Chile; rodrigo.olivares@uv.cl; 6 Faculty of Health Sciences, Universidad Tecnológica Atlántico Mediterráneo (UTAMED), 29590 Malaga, Spain; alejandra.uribe21@gmail.com; 7 Department of Health Research, Icen Cognis, 38370 Tenerife, Spain; 8 Escuela de Kinesiología, Facultad de Salud, Universidad Santo Tomás, Talca 3460000, Chile; eguzmanm@santotomas.cl (E.G.-M.); yenyconchaci@santotomas.cl (Y.C.-C.); 9 Vicerrectoría de Investigación e Innovación, Universidad Arturo Prat, Iquique 1100000, Chile; 10 Centro de Investigación, Desarrollo e Innovación en Salud y Deporte (CIDISAD), Escuela Ciencias del Movimiento Humano y Calidad de Vida (CIEMHCAVI), Universidad Nacional de Costa Rica, Heredia 863000, Costa Rica; drojasv@hotmail.com; 11 Grupo de Investigación en Cultura, Educación y Sociedad, Universidad de la Costa, Barranquilla 080002, Colombia; 12 School of Medicine, Universidad Espíritu Santo, Samborondon 092301, Ecuador; 13 Facultad de Ciencias de la Salud, Universidad Autónoma de Chile, Santiago 7500000, Chile

**Keywords:** hypertension, machine learning, explainable artificial intelligence, SHapley Additive exPlanations, model parsimony, health survey, Chile, complex survey design, prediction model

## Abstract

**Background/Objectives:** Machine learning is increasingly applied to identify hypertension from broad predictor panels, but whether it outperforms a few routine measurements is unclear. We compared machine learning pipelines against three readily obtained variables in a national Chilean survey. **Methods:** We analysed 5516 participants aged 15 years and older from the Chilean National Health Survey 2016–2017 with two valid blood-pressure readings. Hypertension was defined as mean systolic pressure ≥ 140 mmHg or mean diastolic pressure ≥ 90 mmHg or current antihypertensive treatment, the mean taken over the second and third readings. A total of 43 candidate predictors plus 11 laboratory missingness indicators formed 54 model inputs, excluding all blood pressure and hypertension-related variables. Data were partitioned once by census segment. Seventeen algorithms were compared under segment-grouped cross-validation with preprocessing fitted within folds; twelve were tuned identically, nested specifications independently, thresholds locked on development data and intervals derived from segment bootstrap. **Results:** Prevalence was 38.3% unweighted and 29.2% design-weighted. Across seven metrics, the twelve tuned pipelines were closely comparable, and none ranked first on all. On held-out data, the full XGBoost model reached an area under the receiver operating characteristic curve (AUC) of 0.890 (95% confidence interval [CI] 0.872 to 0.906) and the full penalised logistic model 0.882 (0.864 to 0.901). A model containing age, sex and waist-to-height ratio alone reached 0.882 (0.864 to 0.900) under XGBoost and 0.881 (0.861 to 0.898) under penalised logistic regression, differing from the corresponding full model of the nested comparison, which was tuned independently and reached 0.881 under penalised logistic regression and 0.891 under XGBoost, by −0.0004 (−0.0087 to +0.0081) under penalised logistic regression and −0.0084 (−0.0148 to −0.0024) under XGBoost, computed on unrounded values. Removing age cost 0.027 AUC. Findings held under design weighting, in the laboratory subsample and among untreated participants. **Conclusions:** Three routine measurements provided most of the discrimination achieved by fifty-four inputs; the remainder added no detectable increment under a penalised logistic specification and approximately 0.008 AUC under XGBoost. Equivalence was not formally tested, validation was internal, and transportability is undemonstrated.

## 1. Introduction

Hypertension is the single largest modifiable contributor to cardiovascular mortality worldwide, affecting more than one billion adults, and its prevalence has proved resistant to improvement over three decades, and a substantial proportion of affected individuals remain undiagnosed [1,2,3]. Higher usual blood pressure is associated with greater vascular mortality across the attainable range, so even modest population-level shifts are expected to translate into large absolute reductions in mortality [4,5]. The burden falls disproportionately on low- and middle-income settings, where detection rather than treatment is frequently the binding constraint [6].

Contemporary guidelines converge on the same measurement-based diagnostic pathway [7,8,9,10,11], and the modifiable correlates of elevated blood pressure are well characterised: potassium intake [12], dietary pattern [13,14], adiposity [15,16], the distribution of that adiposity [17], physical activity [18,19], alcohol [20] and tobacco [21]. Obesity-related mechanisms and insulin resistance are closely linked to blood pressure regulation [22,23], and socioeconomic position patterns exposure to nearly all of these [24,25].

Against this background, a substantial literature now applies machine learning to identify prevalent or incident hypertension from broad predictor panels [26,27]. Such models routinely report favourable discrimination, but the comparison that determines their practical value—i.e., whether a flexible algorithm operating on dozens of inputs discriminates better than a simple model containing a handful of measurements that are obtained in any clinical encounter—is rarely made. Reporting frameworks for prediction models emphasise precisely this comparison, together with calibration, appropriate handling of the sampling design, and honest accounting of uncertainty [28,29,30,31,32,33].

We therefore examined, in a nationally representative Chilean survey, whether specified machine learning pipelines operating on fifty-four model inputs discriminate prevalent hypertension better than a model containing age, sex, and a single adiposity index. We deliberately excluded every blood pressure measurement and every hypertension-related diagnosis or treatment variable from the predictor set, so that the models could not recover the outcome definition. We further used SHapley Additive exPlanations to describe how each input contributes to model output [34], while treating those contributions as descriptive of the model rather than as evidence about causation.

## 2. Materials and Methods

### 2.1. Reporting and Design

This cross-sectional analysis of secondary survey data is reported according to the Transparent Reporting of a multivariable prediction model for Individual Prognosis Or Diagnosis, Artificial Intelligence extension (TRIPOD+AI) [32], with risk of bias considered against the Prediction model Risk Of Bias Assessment Tool (PROBAST) and its explanation and elaboration document [31,33]. The analysis presented here is a complete reanalysis executed as a single locked pipeline under one random seed (42); every value reported in this manuscript, including all tables, figures, abstract values and the outputs in the Supplementary Materials (Tables S1–S6 and Figures S1–S5), is written directly by that pipeline.

### 2.2. Data Source and Participants

We analysed the Chilean National Health Survey 2016–2017 (Encuesta Nacional de Salud, ENS), a nationally representative, stratified, multistage probability survey of the population aged 15 years and older conducted by the Ministry of Health of Chile and executed by the Pontificia Universidad Católica de Chile [35,36,37]. The survey comprises 6233 respondents distributed across census segments nested within strata. Of these, 5516 (88.5%) had valid second and third blood-pressure readings and constitute the analytic sample. Supplementary Figure S1 presents the participant flow, and the characteristics of the analytic sample are reported in Table 1.

Because the survey includes participants from 15 years of age, we refer throughout to “participants aged 15 years and older” rather than to adults. The analytic sample includes 214 participants aged 15 to 17 years (3.9%); a sensitivity analysis restricted to those aged 18 and over is reported.

### 2.3. Blood-Pressure Measurement and Outcome Definition

Blood pressure was measured by trained and certified examiners using a validated automated oscillometric device. Cuff size was selected from the measured arm circumference. Participants rested seated for at least five minutes before the first reading, and three readings were obtained at intervals of at least one minute. Quality control comprised centralised examiner training and certification, standardised field protocols, and periodic equipment calibration [35,37].

Hypertension was defined as mean systolic blood pressure ≥140 mmHg or mean diastolic blood pressure ≥90 mmHg or current use of antihypertensive medication, the mean being taken over the second and third readings and the first reading being discarded. The disjunction is inclusive: a participant meeting any single criterion is classified as hypertensive. The same definition, in the same wording, is used in the Abstract and in the Results. Current antihypertensive treatment was operationalised as an affirmative response to survey item m2p1, administered by the examiner at the physical-examination visit. Applying this definition yields 2114 hypertensive participants (38.32%). For transparency, we note that the alternative interview-based item on current treatment would yield 2038 cases (36.95%); the examination-based item was used throughout.

For sensitivity analyses, we additionally derived a four-level phenotype: untreated normotensive, untreated hypertensive, treated with controlled measured blood pressure, and treated with uncontrolled measured blood pressure.

### 2.4. Candidate Predictors and Model Inputs

A total of 43 candidate predictors were specified a priori across 7 domains: demography and socioeconomic position (8), adiposity (4), physical activity (1), diet (14), tobacco (2), psychosocial factors (3), and laboratory measurements (11). Supplementary Tables S1a and S1b enumerate every model input individually with its source variable in the survey microdata, measurement scale, coding, completeness in the analytic sample, and encoding.

Eleven binary indicators, one for each laboratory variable, recorded whether that measurement was observed. Forty-three candidate predictors plus eleven missingness indicators therefore constitute fifty-four model inputs. We use “model inputs” rather than “variables” throughout, because the eleven indicators are derived from the predictors rather than independent of them.

Estimated glomerular filtration rate was computed from serum creatinine using the race-free 2021 Chronic Kidney Disease Epidemiology Collaboration (CKD-EPI) equation [38]. The survey supplies the urinary sodium-to-creatinine and potassium-to-creatinine ratios as measured variables; the urinary sodium-to-potassium ratio, the triglyceride–glucose index and the total-to-high-density lipoprotein (HDL) cholesterol ratio were derived from their components. All three urinary variables were retained as separate model inputs and are listed individually in Supplementary Table S1a. Waist-to-height ratio was computed from measured waist circumference and standing height.

No blood-pressure reading, no self-reported hypertension diagnosis and no antihypertensive treatment variable entered the predictor set. This exclusion was enforced programmatically by an assertion in the pipeline that fails if any banned variable appears among the model inputs.

Two candidate laboratory measurements available in the survey were not retained. Serum uric acid was not measured in this survey wave. Microalbuminuria was measured but is ascertained only within a subset of the laboratory phase, so retaining it would have introduced a second missingness mechanism, structurally distinct from the planned laboratory subsampling that the eleven indicators are designed to carry, and it was therefore excluded. A completeness threshold of 65% was applied to the source laboratory measurements; the triglyceride–glucose index, at 64.9% complete, falls marginally below that threshold and was retained because its completeness is determined entirely by that of its two source measurements, both of which exceed it. Supplementary Table S1a reports completeness for every candidate predictor individually.

The variable supplying low-density lipoprotein (LDL) cholesterol carries the official label “COLESTEROL-LDL DIRECTO” although its variable name indicates a calculated value. We resolved this by computation: the reported values reproduce the Friedewald estimate from total cholesterol, HDL cholesterol, and triglycerides to within 0.4 mg/dL on average (r = 0.9999 among the 3631 participants with triglycerides below 400 mg/dL), so the values are Friedewald-calculated and the official label is inaccurate. We report them as calculated.

### 2.5. Missing Data

Numeric inputs were imputed to the training-fold median and nominal inputs to the training-fold mode. Single imputation was used rather than multiple imputation because the imputer forms part of a locked prediction pipeline that must be applied unchanged to unseen participants; the resulting understatement of imputation uncertainty is acknowledged, and the complete case analysis within the laboratory subsample reported in Section 3.7 serves as the corresponding sensitivity analysis [39]. The imputer was fitted inside each cross-validation training fold and applied unchanged to the corresponding validation fold and to the held-out partition, so that no information crossed the partition boundary. Laboratory missingness arises predominantly from planned subsampling rather than from non-response and is carried explicitly by the eleven indicators. Complete-case and inverse-probability-weighted analyses within the laboratory subsample are reported as sensitivity analyses.

### 2.6. Partitioning

The analytic sample was partitioned once, before any model was fitted, into a development set of 4104 participants in 810 census segments and a held-out set of 1412 participants in 271 census segments. Partitioning was performed at the level of the census segment, so that no segment contributes participants to both sets and geographical clustering cannot produce optimistic performance [30]. Balance between partitions is reported in Supplementary Table S2. This split was generated once and written to disk; every subsequent analysis in this manuscript uses that same split.

### 2.7. Algorithm Comparison and Preprocessing

Seventeen supervised algorithms were compared under stratified five-fold cross-validation grouped by census segment. All preprocessing was fitted inside each training fold. Two representations were used, specified in advance by algorithm class: tree ensembles received native integer codes with median imputation, whereas scale-sensitive algorithms—logistic regression, both discriminant analyses, naive Bayes, k-nearest neighbours, the support vector machine and the multilayer perceptron—received one-hot encoding of the three nominal inputs together with standardisation of continuous and ordinal inputs. Evaluating all algorithms on a single representation, as in our previous analysis, disadvantages preprocessing-sensitive methods and we do not do so here. No class-imbalance correction was applied, since such corrections degrade calibration without improving discrimination in settings of this kind [40].

### 2.8. Hyperparameter Optimisation

Two distinct gradient-boosting implementations appear in this manuscript and are named consistently throughout. “Gradient boosting (sklearn)” denotes the scikit-learn GradientBoostingClassifier, one of the seventeen candidates in Table 2 and one of the twelve tuned pipelines in Table 3. “XGBoost” denotes the extreme gradient-boosting implementation, which is the model carried into the held-out evaluation, the nested comparisons and the attribution analysis, and which is referred to as XGBoost in every table and figure rather than by the generic term. Two cross-validated estimators are reported and are not interchangeable. Table 2 reports the mean and standard deviation of each metric across the five folds, which is the quantity used for algorithm selection. Table 3 reports metrics computed once from the pooled out-of-fold predicted probabilities, because threshold-dependent metrics require a single pooled prediction vector and a single threshold. Each quantity appears exactly once in this submission; performance of the tuned pipelines is reported only in Table 3 and is not repeated in the Supplementary Material.

The selection rule was prespecified: every algorithm whose default cross-validated area under the receiver operating characteristic curve (AUC) fell within 0.02 of the leading default models proceeded to tuning. The leading default model attained 0.877, so the threshold was 0.857; twelve of the seventeen algorithms met the criterion and all twelve were tuned. None meeting the criterion were omitted and none failing it were included. Tuning used randomised search over the same cross-validation folds, with between 8 and 30 configurations per algorithm according to computational cost. Complete search spaces and selected values are given in Supplementary Table S3.

### 2.9. Definition of the Nested Specifications

A total of 5 nested specifications were evaluated: parsimonious (age, sex and waist-to-height ratio [WHtR]), anthropometric (those 3 plus body mass index, waist circumference and arm circumference), non-laboratory (32 inputs), full (54 inputs) and full-without-age (53 inputs). Each was tuned independently on the development data alone, over the same cross-validation folds and the same search spaces used in the 12-algorithm comparison, and without any reference to the held-out partition. Hyperparameters optimised for the full input set were not carried into the nested models. The randomised-search budget for these specifications was 15 configurations under the XGBoost search space and 10 under the penalised logistic search space, against 30 for the corresponding models in Table 3; this smaller budget, and not any difference in data or in folds, is why the full XGBoost model reported in Table 4 attains 0.890 while the independently tuned full XGBoost model reported in Table 5 attains 0.8905 before rounding, which is shown as 0.891 in Table 5; the two fits differ in the fourth decimal place.

### 2.10. Operating Threshold

The operating threshold for each model was obtained by maximising Youden’s J over the cross-validated out-of-fold predicted probabilities in the development data and was written to disk before the held-out partition was accessed. Each nested specification carries its own independently derived and locked threshold. No threshold was selected or adjusted using held-out data.

### 2.11. Evaluation and Uncertainty

Discrimination was quantified by the area under the receiver operating characteristic curve and by average precision, reported as AUC-PR in Table 2, Table 3 and Table 4; calibration by the calibration slope, the calibration intercept and the expected calibration error over deciles of predicted risk [29]; and the overall accuracy of the probabilities by the Brier score. Sensitivity, specificity, and predictive values were computed at the locked threshold.

All interval estimates were obtained by bootstrap resampling of census segments with replacement, participants moving as intact clusters, over 2000 replicates. Resampling individuals would ignore within-segment dependence and yield intervals that are too narrow; we do not do so anywhere in this manuscript. Paired differences between models were computed within each bootstrap replicate.

### 2.12. Handling of the Survey Design and Planned Sensitivity Analyses

The primary analysis is unweighted, on the grounds that the objective is model development rather than population estimation, and all primary claims are anchored to the analytic sample. A fully design-weighted evaluation is reported alongside it, using the survey expansion factor together with strata and primary sampling units; this factor reproduces the published weighted prevalence of 29.2% exactly. This design-weighted evaluation addresses discrimination and the Brier score; calibration, predictive values and all threshold-dependent quantities are prevalence-dependent and therefore remain explicitly anchored to the analytic sample. Four further sensitivity analyses are reported: restriction to participants with fully observed biochemistry, with and without laboratory-phase weights; restriction to untreated participants with the outcome defined solely by measured blood pressure; restriction to participants aged 18 and over; and a comparison of single adiposity indices.

### 2.13. Attribution Analysis

Contributions of individual inputs to model output were quantified by SHapley Additive exPlanations (SHAP) computed with TreeExplainer (shap 0.52.0) on the held-out partition [34]. A single computation was performed, and the resulting array is the sole source of every SHAP value reported in this manuscript, in the tables, in the figures and in the text.

The model expected value, against which all contributions are referenced, was −0.4604 on the log-odds scale. A single fitted model and this single expected value were used for every reported attribution, including all stratified summaries, so that strata are directly comparable.

Exact pairwise SHAP interaction values were computed for the twelve highest-ranked inputs. Because exact interaction computation scales quadratically in the number of inputs, these values were obtained on a random sample of 400 held-out participants drawn under the same seed; all other attribution values reported in this manuscript derive from the single full-partition computation described above. Rank stability was assessed by refitting the model on 25 segment-level bootstrap resamples of the development data and recomputing attributions each time. To test whether the fitted relationship is approximately additive, a depth-one XGBoost model, which cannot represent interactions by construction, was compared with the tuned interaction-enabled model.

SHAP values quantify each input’s contribution to the model’s output for a given participant. They carry no causal interpretation. In a cross-sectional design this restriction applies with particular force to glucose, renal function, lipids and socioeconomic position, each of which may be a consequence of hypertension or of its treatment rather than an antecedent.

Direction of association is reported only where interpretable, under an explicit rule: nominal inputs are excluded; for ordinal and continuous inputs, a direction is reported only when the sign of the Spearman coefficient between input value and SHAP value agrees with the sign of the change in mean SHAP value across at least three of the four adjacent quintile transitions. Inputs failing this criterion are marked non-monotonic rather than assigned a direction. Binary inputs, including all eleven missingness indicators, are reported as a contrast between their two levels.

### 2.14. Sample Size

The required sample size for model development was verified following Riley and colleagues for binary outcomes [41,42]. Assumptions were as follows: anticipated outcome proportion 0.383; 66 parameters after encoding (fifty-four model inputs, with one-hot expansion of three nominal variables adding twelve columns); assumed Cox–Snell R^2^ of 0.15 against a maximum attainable value of 0.74 at this prevalence; and target shrinkage 0.90. The resulting requirement is 3620 participants, and the criterion for estimating the outcome proportion to within 0.05 requires 363. The development partition contains 4104 participants with 1590 events, giving 24.1 events per parameter and exceeding the binding requirement.

### 2.15. Software

Analyses were performed in Python 3.12 with scikit-learn 1.8.0, XGBoost 3.4.1, LightGBM 4.7.0, CatBoost 1.2.10, shap 0.52.0, statsmodels 0.14.6, pandas 3.0.2, NumPy 2.4.4, SciPy 1.17.1 and Matplotlib 3.10.9. The random seed was fixed at 42 throughout. The complete pipeline is provided as Supplementary Material.

## 3. Results

### 3.1. Participants

Of 6233 survey respondents, 5516 (88.5%) had two valid blood pressure readings and formed the analytic sample. Hypertension was present in 2114 participants, a crude prevalence of 38.32% and a design-weighted prevalence of 29.21%. The four-level phenotype comprised 3402 untreated normotensive participants (61.7%), 773 treated with controlled measured blood pressure (14.0%), 767 treated with uncontrolled measured blood pressure (13.9%), and 574 untreated hypertensive participants (10.4%). Thus, 27.9% of the sample is classified as hypertensive on the basis of treatment, and half of those have controlled measured blood pressure.

### 3.2. Balance Between Partitions

The development and held-out partitions were well balanced. Standardised differences were 0.021 for age, 0.038 for body mass index, 0.046 for waist circumference, 0.050 for waist-to-height ratio, −0.013 for fasting glucose, −0.005 for sex, and −0.034 for the outcome, all far below the conventional threshold of 0.10. The single nominally significant difference was urban residence (84.8% versus 82.2%; standardised difference 0.070; *p* = 0.024), an expected consequence of randomising intact geographical clusters and of negligible magnitude. The full balance table is given in Supplementary Table S2.

### 3.3. Comparison of Seventeen Algorithms at Default Settings

Under segment-grouped cross-validation with default hyperparameters, cross-validated AUC ranged from 0.699 for a single decision tree to 0.877 for logistic regression (Table 2). Logistic regression ranked first, followed by linear discriminant analysis, gradient boosting (sklearn), and CatBoost. The multilayer perceptron, quadratic discriminant analysis, naive Bayes, k-nearest neighbours, and single decision tree performed appreciably worse. This spread is incompatible with any claim that all seventeen algorithms performed equivalently, and no such claim is made.

**Table 2:** Cross-validated performance of seventeen algorithms at default settings in the development set, under stratified five-fold cross-validation grouped by census segment. SD is the standard deviation of AUC-ROC across folds. AUC-ROC, area under the receiver operating characteristic curve; AUC-PR, area under the precision–recall curve; RBF, radial basis function; RMSE, root mean squared error of the predicted probabilities. Threshold-dependent metrics use a probability cut-off of 0.5. “Gradient boosting (sklearn)” is the scikit-learn GradientBoostingClassifier and is distinct from XGBoost, as defined in Section 2.8; “penalised logistic regression” denotes the regularised logistic model used throughout.

Algorithm	AUC-ROC	SD	AUC-PR	F1	Balanced Accuracy	Brier	RMSE
Penalised logistic regression	0.877	0.008	0.802	0.733	0.783	0.140	0.374
Linear discriminant analysis	0.876	0.009	0.801	0.735	0.784	0.141	0.375
Gradient boosting (sklearn)	0.875	0.008	0.800	0.733	0.782	0.140	0.374
CatBoost	0.875	0.011	0.802	0.738	0.786	0.141	0.375
Extremely randomised trees	0.869	0.013	0.788	0.711	0.767	0.148	0.385
Support vector machine (RBF)	0.868	0.015	0.788	0.723	0.775	0.144	0.380
Histogram gradient boosting	0.867	0.009	0.786	0.726	0.777	0.148	0.385
AdaBoost	0.866	0.012	0.782	0.731	0.780	0.183	0.427
LightGBM	0.866	0.010	0.781	0.721	0.772	0.149	0.386
Random forest	0.865	0.012	0.780	0.727	0.777	0.146	0.383
Bagging	0.860	0.010	0.769	0.723	0.774	0.148	0.385
XGBoost	0.859	0.008	0.775	0.709	0.764	0.161	0.402
Quadratic discriminant analysis	0.830	0.018	0.711	0.694	0.751	0.181	0.425
Multilayer perceptron	0.813	0.014	0.704	0.670	0.731	0.227	0.476
Gaussian naive Bayes	0.806	0.014	0.703	0.625	0.627	0.406	0.637
k-nearest neighbours	0.773	0.022	0.636	0.612	0.693	0.190	0.436
Decision tree	0.699	0.017	0.540	0.633	0.699	0.288	0.537

### 3.4. Hyperparameter Optimisation and Multi-Metric Comparison

Twelve algorithms fell within 0.02 AUC of the leading default model and were tuned under an identical protocol. Because discrimination alone is an insufficient basis on which to judge equivalence, we recomputed seven metrics for every tuned pipeline from the same segment-grouped cross-validation folds used for selection, spanning discrimination, threshold-dependent classification and probability accuracy (Table 3). Complete search spaces and selected hyperparameters are given in Supplementary Table S3.

On discrimination, the twelve pipelines are tightly clustered: cross-validated AUC-ROC ranges from 0.865 to 0.877, representing a spread of 0.012. This spread is of the same order as the fold-to-fold standard deviation observed at default settings, which ranged from 0.008 to 0.015 across these twelve algorithms (Table 2), and is smaller than that standard deviation for three of them. AUC-PR spans 0.026, F1 0.015, balanced accuracy 0.012, and a Matthews correlation coefficient of 0.020; all five spreads are computed on the unrounded values. On these five axes, the pipelines are closely comparable, although we do not claim that the between-algorithm spread is uniformly smaller than within-algorithm sampling variability.

Probability accuracy separates them more clearly. The Brier score ranges from 0.139 to 0.173, and the root mean squared error from 0.372 to 0.416, spreads of 0.035 and 0.044 computed on the unrounded values, respectively. This is driven almost entirely by AdaBoost, whose Brier score of 0.1731 is approximately 25% worse than that of the leading pipelines despite a cross-validated AUC-ROC of 0.871 that places it tenth of twelve. Excluding AdaBoost, the Brier range narrows to 0.008. Discrimination and probability accuracy are therefore not interchangeable summaries, and reporting AUC alone would have concealed a material deficiency in one of the twelve candidates.

No pipeline ranked first on every metric. Penalised logistic regression ranked first on AUC-ROC, F1, balanced accuracy and the Matthews correlation coefficient, second on AUC-PR and third on the Brier score; linear discriminant analysis ranked first on AUC-PR but eighth on the Brier score; XGBoost ranked first on the Brier score and root mean squared error but second on AUC-ROC. Under any reasonable weighting of these criteria, the leading pipelines are indistinguishable, and we therefore identify no best-performing algorithm. Formal equivalence was not tested for any of them.

**Table 3:** Multi-metric cross-validated performance of the twelve tuned algorithms in the development set, computed from the same segment-grouped folds used for selection. Threshold-dependent metrics (F1, balanced accuracy, MCC) are evaluated at each pipeline’s own Youden threshold derived from its out-of-fold predicted probabilities, shown in the final column; no threshold was taken from the held-out partition. MCC, Matthews correlation coefficient; Balanced acc., balanced accuracy; RBF, radial basis function; RMSE, root mean squared error of the predicted probabilities. Rows are ordered by AUC-ROC; the ordering differs by metric, and no pipeline ranks first on all of them.

Algorithm	AUC-ROC	AUC-PR	F1	Balanced Acc.	MCC	Brier	RMSE	Threshold
Penalised logistic regression	0.877	0.801	0.760	0.803	0.590	0.139	0.373	0.324
XGBoost	0.877	0.800	0.757	0.799	0.584	0.139	0.372	0.360
CatBoost	0.877	0.801	0.755	0.799	0.582	0.139	0.372	0.365
Linear discriminant analysis	0.877	0.803	0.757	0.799	0.583	0.140	0.374	0.304
Histogram gradient boosting	0.876	0.800	0.755	0.798	0.581	0.139	0.373	0.371
Support vector machine (RBF)	0.876	0.800	0.756	0.799	0.585	0.140	0.374	0.359
LightGBM	0.876	0.798	0.755	0.798	0.581	0.139	0.373	0.347
Gradient boosting (sklearn)	0.875	0.797	0.754	0.796	0.578	0.140	0.374	0.327
Extremely randomised trees	0.873	0.791	0.749	0.793	0.574	0.142	0.377	0.429
AdaBoost	0.871	0.792	0.750	0.794	0.574	0.173	0.416	0.463
Random forest	0.869	0.782	0.749	0.792	0.570	0.143	0.378	0.376
Bagging	0.865	0.777	0.746	0.791	0.572	0.146	0.383	0.442

### 3.5. Held-Out Performance

On the held-out partition of 1412 participants in 271 previously unseen census segments (524 events), the full XGBoost model achieved AUC 0.890 (95% confidence interval (CI) 0.872 to 0.906), CatBoost 0.889 (95% CI 0.871 to 0.906), and the full penalised logistic model 0.882 (95% CI 0.864 to 0.901). Paired differences obtained within bootstrap replicates were −0.008 (95% CI −0.032 to +0.017) for the penalised logistic model versus XGBoost and 0.000 (95% CI −0.024 to +0.026) for XGBoost versus CatBoost. No model was superior to any other by any criterion applied.

**Figure 1:**
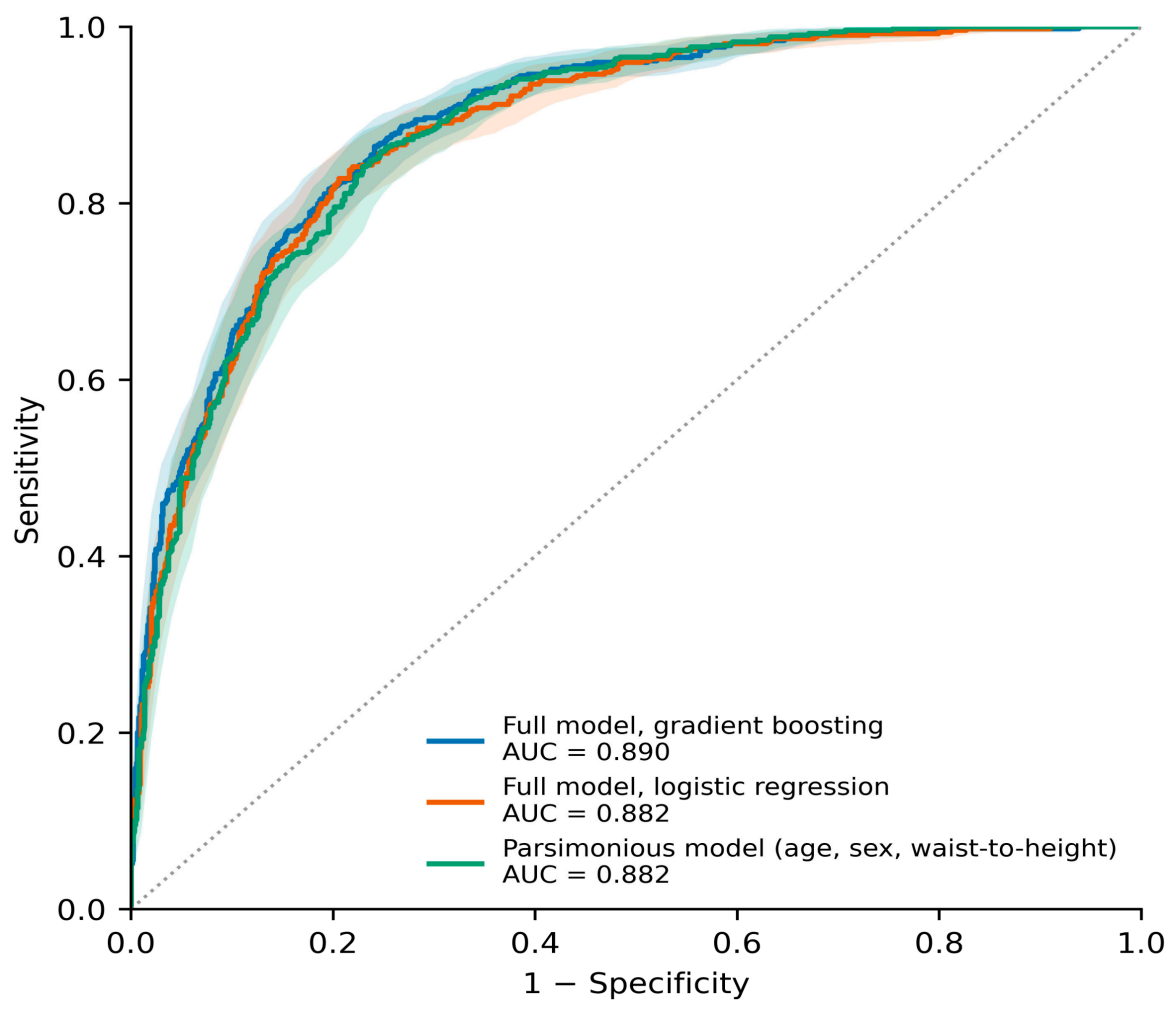
Receiver operating characteristic curves on the held-out partition (*n* = 1412; 524 events) for three models: the full XGBoost model, the full penalised logistic model, and the parsimonious XGBoost model containing age, sex and waist-to-height ratio. The vertical axis is sensitivity, and the horizontal axis is one minus specificity. The dotted diagonal denotes the line of no discrimination (area under the curve 0.5). Shaded regions are 95% bootstrap bands obtained by resampling the 271 held-out census segments, so their width reflects between-segment rather than between-participant variability. The near-coincidence of the parsimonious and full curves across the operating range is the graphical form of the difference of 0.0084 AUC reported in Table 5.

**Figure 2:**
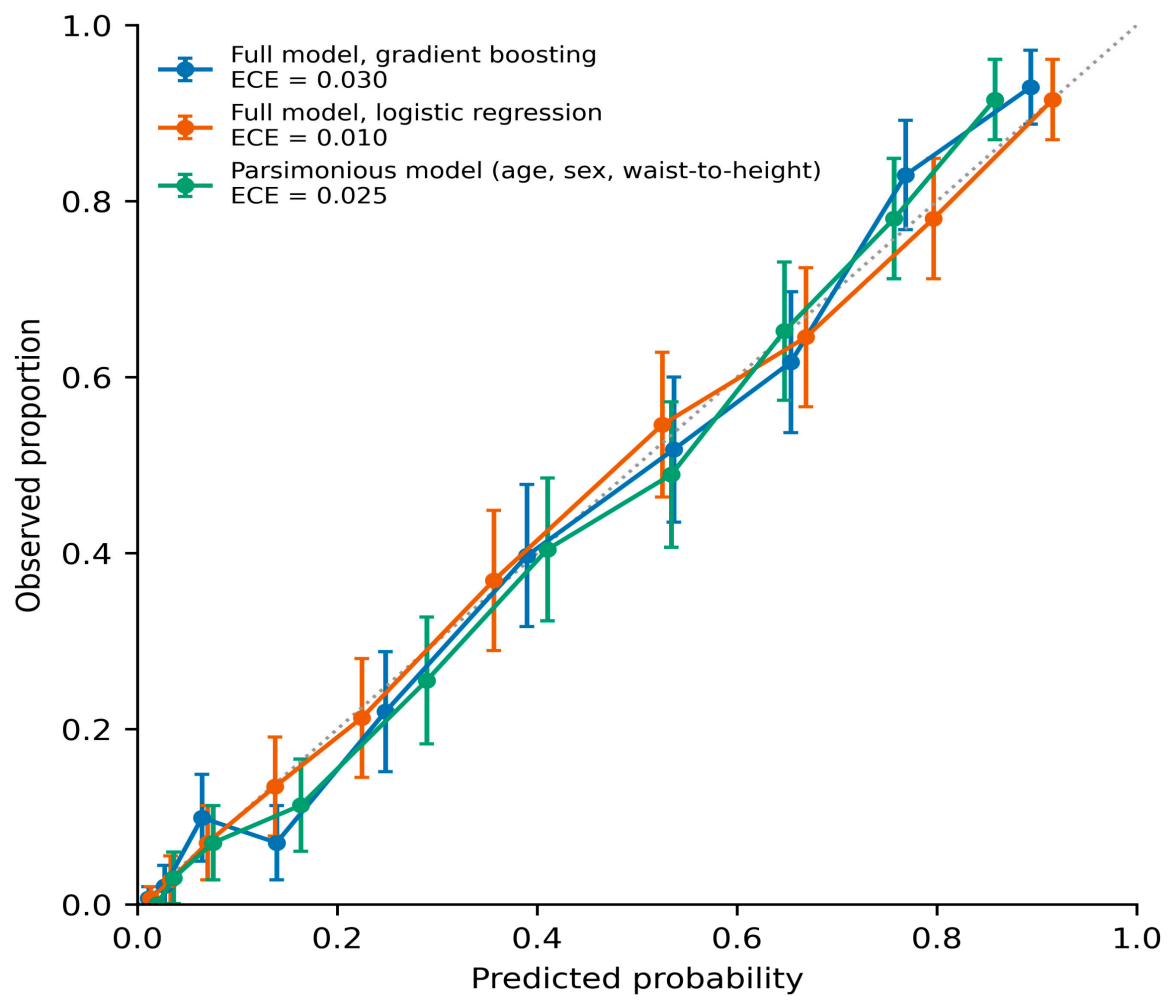
Calibration of the same three models as Figure 1 on the held-out partition. Predicted probabilities are grouped into deciles; the horizontal axis is the mean predicted probability within each decile and the vertical axis the observed proportion of hypertensive participants, with 95% binomial intervals. The diagonal denotes perfect agreement between predicted and observed risk, and points below it indicates over-prediction. Expected calibration error (ECE) is the mean absolute deviation between the two across deciles and is reported as a point estimate only, for the reason given in the note to Table 4. The held-out values are 0.030 for the full XGBoost model, 0.010 for the full penalised logistic model, and 0.025 for the parsimonious XGBoost model. The first two are the values reported in Table 4; the third belongs to the parsimonious specification of Table 5, which is not among the models in Table 4, whose third model is CatBoost.

At their independently locked thresholds, the XGBoost model achieved sensitivity of 0.855 (95% CI 0.824 to 0.884) and specificity of 0.761 (95% CI 0.731 to 0.790), and the logistic model achieved a sensitivity of 0.851 (95% CI 0.819 to 0.883) and specificity of 0.755 (95% CI 0.724 to 0.783). Calibration was close to ideal for both: calibration slope was 1.000 (95% CI 0.904 to 1.117) for the penalised logistic model and 1.094 (95% CI 0.980 to 1.228) for XGBoost, with intercepts indistinguishable from zero. Receiver operating characteristic curves with bootstrap bands are shown in Figure 1 and calibration curves in Figure 2.

**Table 4:** Held-out performance of the three principal full models (*n* = 1412; 524 events; 271 census segments), each evaluated at its own operating threshold derived from development out-of-fold predictions and locked before evaluation. All intervals are 95% bootstrap intervals obtained by resampling census segments, except for the expected calibration error, which is reported as a point estimate only: binned expected calibration error is a non-negative statistic that is biased upward under resampling (bootstrap medians 0.035, 0.032 and 0.025 against held-out values of 0.030, 0.021 and 0.010), so a percentile interval would not be centred on the estimate and is not reported.

Metric	XGBoost	CatBoost	Penalised Logistic Regression
AUC-ROC	0.890 (0.872, 0.906)	0.889 (0.871, 0.906)	0.882 (0.864, 0.901)
AUC-PR	0.825 (0.791, 0.855)	0.825 (0.791, 0.855)	0.812 (0.780, 0.844)
Brier score	0.129 (0.120, 0.140)	0.129 (0.119, 0.140)	0.133 (0.122, 0.144)
Sensitivity	0.855 (0.824, 0.884)	0.855 (0.823, 0.885)	0.851 (0.819, 0.883)
Specificity	0.761 (0.731, 0.790)	0.766 (0.736, 0.796)	0.755 (0.724, 0.783)
Positive predictive value	0.679 (0.645, 0.714)	0.683 (0.647, 0.718)	0.672 (0.636, 0.704)
Negative predictive value	0.899 (0.878, 0.919)	0.899 (0.877, 0.921)	0.896 (0.873, 0.918)
Calibration slope	1.094 (0.980, 1.228)	1.085 (0.980, 1.212)	1.000 (0.904, 1.117)
Calibration intercept	−0.017 (−0.140, 0.111)	−0.019 (−0.152, 0.111)	−0.024 (−0.160, 0.106)
Expected calibration error	0.030	0.021	0.010

### 3.6. Nested Specifications

Each nested specification was tuned independently (Table 5). Under logistic regression, the parsimonious model containing only age, sex and waist-to-height ratio was statistically indistinguishable from the full fifty-four-input model, the paired difference being −0.0004 (95% CI −0.0087 to +0.0081). Under XGBoost, the full model was modestly but detectably better, the difference being −0.0084 (95% CI −0.0148 to −0.0024).

The increment attributable to the additional inputs is therefore small and depends on the specification. The interval for the logistic comparison is compatible with the fifty-one additional inputs slightly degrading or slightly improving discrimination, the largest improvement compatible with the data being approximately 0.008 AUC. The interval for the XGBoost comparison excludes zero, and the largest increment compatible with the data is approximately 0.015 AUC.

Removing age alone from the full input set cost 0.027 AUC under XGBoost (95% CI −0.0349 to −0.0179) and 0.028 under logistic regression (95% CI −0.0369 to −0.0195)—an order of magnitude more than the entire remaining panel contributes. At the same time, the age-free model still reached AUC 0.864 (0.844 to 0.883), so the fifty-three non-age inputs retain substantial joint discrimination in the absence of age.

**Table 5:** Held-out performance of nested specifications, each tuned independently on the development data with an identical protocol. Paired differences against the corresponding full model were computed within bootstrap replicates resampling census segments. Each specification, including the full one, was tuned independently, so the full XGBoost model here is a separately optimised fit, and its AUC, 0.8905 before rounding and shown as 0.891 in the table, differs slightly from that of the XGBoost model in Table 4 (0.890), which was tuned within the twelve-algorithm comparison. Calib. slope, calibration slope; Logistic reg., penalised logistic regression.

Specification	Inputs	Algorithm	AUC (95% CI)	Brier (95% CI)	Calib. Slope	ΔAUC vs. Full Model (95% CI)
Parsimonious	3	XGBoost	0.882 (0.864, 0.900)	0.135 (0.125, 0.146)	1.168	−0.0084 (−0.0148, −0.0024)
Anthropometric	6	XGBoost	0.882 (0.863, 0.899)	0.135 (0.126, 0.146)	1.177	−0.0084 (−0.0147, −0.0024)
Non-laboratory	32	XGBoost	0.884 (0.865, 0.901)	0.134 (0.124, 0.144)	1.116	−0.0067 (−0.0117, −0.0017)
Full	54	XGBoost	0.891 (0.872, 0.908)	0.129 (0.119, 0.140)	1.138	reference
Full without age	53	XGBoost	0.864 (0.844, 0.883)	0.145 (0.135, 0.155)	1.163	−0.0266 (−0.0349, −0.0179)
Parsimonious	3	Logistic reg.	0.881 (0.861, 0.898)	0.135 (0.124, 0.147)	1.082	−0.0004 (−0.0087, +0.0081)
Anthropometric	6	Logistic reg.	0.879 (0.861, 0.897)	0.136 (0.126, 0.147)	1.102	−0.0019 (−0.0097, +0.0061)
Non-laboratory	32	Logistic reg.	0.880 (0.862, 0.898)	0.136 (0.125, 0.146)	1.045	−0.0014 (−0.0062, +0.0034)
Full	54	Logistic reg.	0.881 (0.861, 0.899)	0.134 (0.123, 0.146)	1.003	reference
Full without age	53	Logistic reg.	0.853 (0.833, 0.873)	0.150 (0.139, 0.161)	0.998	−0.0283 (−0.0369, −0.0195)

### 3.7. Survey Design and Sensitivity Analyses

Design weighting did not materially alter discrimination. Weighted held-out AUC was 0.891 (95% CI 0.851 to 0.923) for XGBoost and 0.883 (0.846 to 0.914) for the penalised logistic model, against unweighted values of 0.890 and 0.882. Weighted Brier scores were lower (0.115 and 0.119 against 0.129 and 0.133), as expected given the lower weighted prevalence. Discrimination is therefore insensitive to the sampling design, whereas absolute risk and threshold-dependent quantities are prevalence-dependent and remain anchored to the analytic sample.

Participants with and without observed biochemistry were almost identically distributed: standardised differences were 0.014 for age, −0.024 for body mass index, −0.031 for waist-to-height ratio and −0.015 for hypertension itself. This is the signature of subsampling by design and indicates that the laboratory missingness indicators cannot plausibly encode an informative selection process.

Restricting both development and evaluation to participants with fully observed biochemistry (held-out *n* = 860; 317 events), the full model reached AUC 0.883 (0.860 to 0.905) and the non-laboratory model 0.872 (0.846 to 0.895). With laboratory-phase weights applied, the values were 0.890 and 0.882. The increment attributable to the laboratory panel is therefore approximately 0.01 AUC whether estimated with imputation in the complete sample or without imputation in the subsample; the parsimony finding is not an artefact of median imputation.

Among untreated participants only, with the outcome defined solely by measured blood pressure (held-out *n* = 1040; 152 events; prevalence 14.6%), the full XGBoost model reached AUC 0.825 (0.790 to 0.857) and the penalised logistic model 0.813 (0.777 to 0.846). Discrimination was lower than in the complete sample, as expected when the treated stratum—which is older and metabolically distinct—was removed, but remained substantial, and the structure of the findings was unchanged.

Restricting to participants aged 18 years and over (held-out *n* = 1351) yielded an AUC of 0.883 (0.865 to 0.901) for XGBoost and 0.875 (0.855 to 0.894) for the penalised logistic model. No conclusion depends on the inclusion of the 214 participants aged 15 to 17.

Single adiposity indices, each entered alongside age and sex in a penalised logistic model, performed interchangeably (Table 6). Waist-to-height ratio was nominally highest, but the intervals overlap almost completely, and this ordering should not be relied upon.

Lipid-lowering treatment was reported by 18.4% of hypertensive participants and 4.2% of non-hypertensive participants with lipid measurements, a fourfold difference. Mean total cholesterol among those on treatment was 179.8 mg/dL at a mean age of 63.2 years, against 181.5 mg/dL at a mean age of 47.7 years among those untreated.

### 3.8. Subgroup Performance

**Figure 3:**
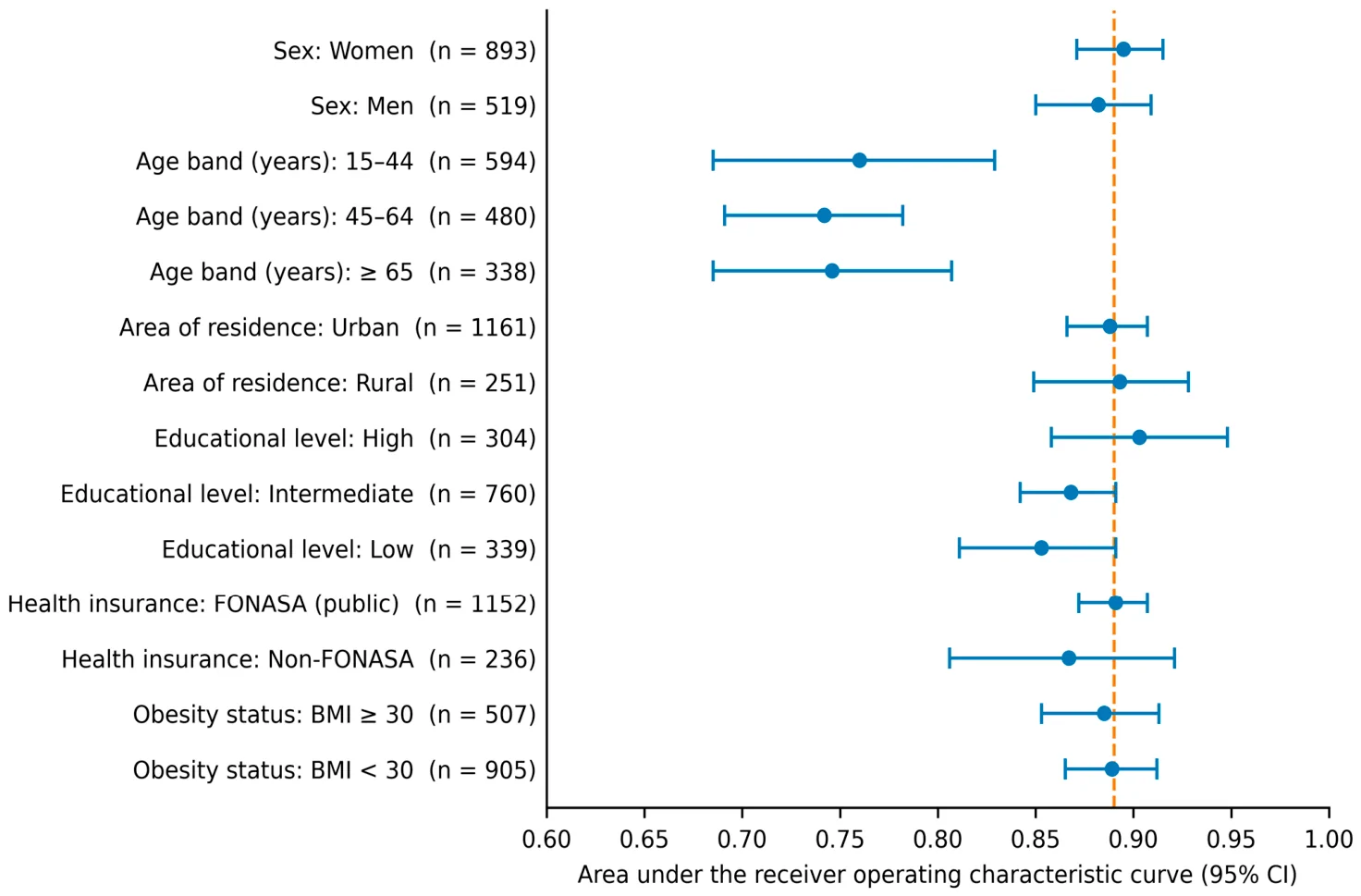
Held-out discrimination of the full XGBoost model within strata, with 95% bootstrap intervals obtained by resampling census segments. The dashed vertical line marks the overall held-out estimate. No pairwise contrast reached significance.

Discrimination was homogeneous across all examined strata (Figure 3 and Table 7). No pairwise contrast approached significance: sex *p* = 0.48; age bands *p* = 0.62 to 0.92; area of residence *p* = 0.78; educational level *p* = 0.11 to 0.57; health insurance (Fondo Nacional de Salud (FONASA) versus non-FONASA) *p* = 0.48; obesity status *p* = 0.86. The educational gradient suggested by point estimates in our previous analysis is not supported once uncertainty is accounted for.

Within age bands, AUC was 0.760 (0.685 to 0.829) at 15 to 44 years, 0.742 (0.691 to 0.782) at 45 to 64 years and 0.746 (0.685 to 0.807) at 65 years and over, against 0.890 overall. This reduction is the expected consequence of conditioning on a dominant predictor: restricting the range of age removes case-mix heterogeneity and lowers AUC without implying any loss of model validity. We do not interpret it as evidence that overall performance is an artefact of age, particularly since the age-free model retains AUC 0.864.

### 3.9. Contributions of Individual Inputs to Model Output

**Figure 4:**
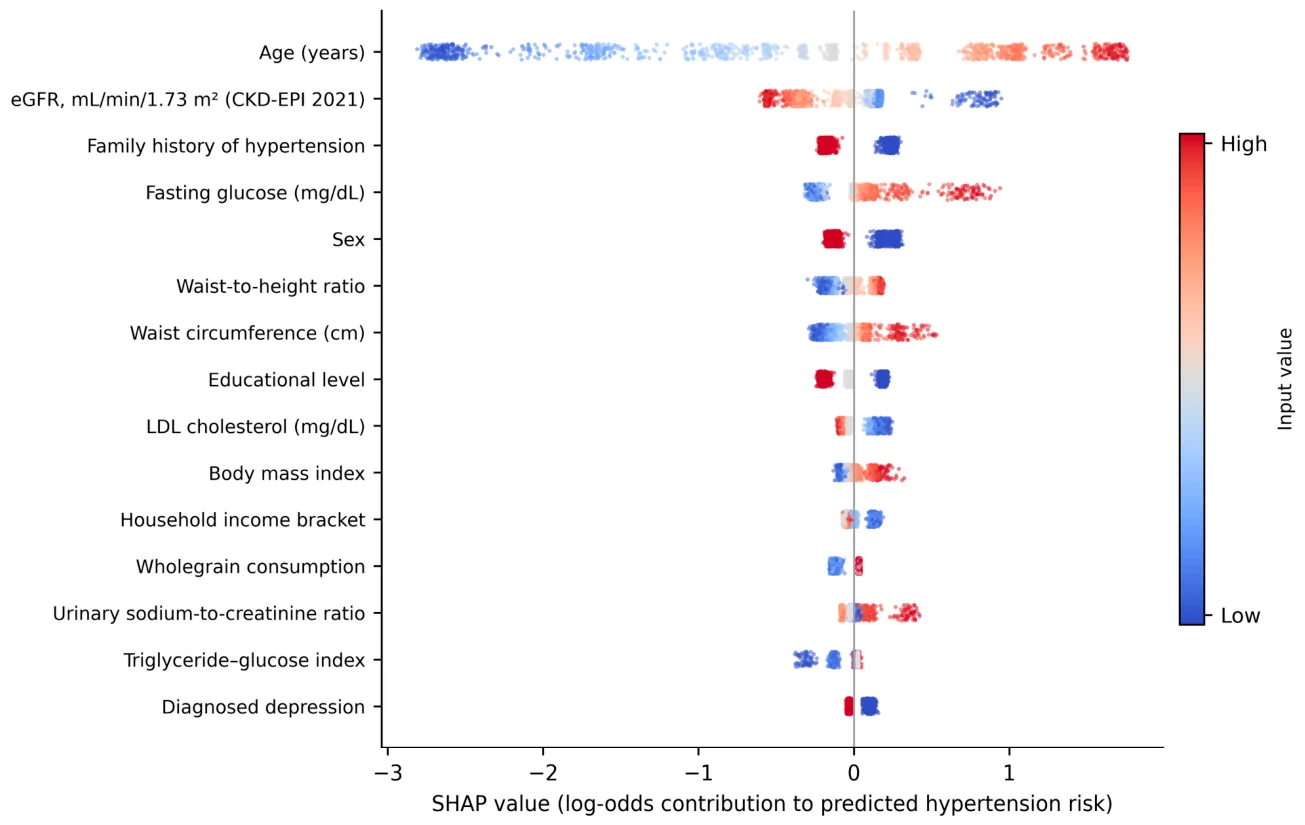
Distribution and direction of individual contributions to the output of the full XGBoost model for the fifteen highest-ranked inputs on the held-out partition. Each point is one participant; horizontal position is the SHAP value on the log-odds scale, and colour encodes the percentile of the input value. This figure is generated from the same single attribution computation as Table 8 and Figure 5, Figure 6 and Figure 7.

**Figure 5:**
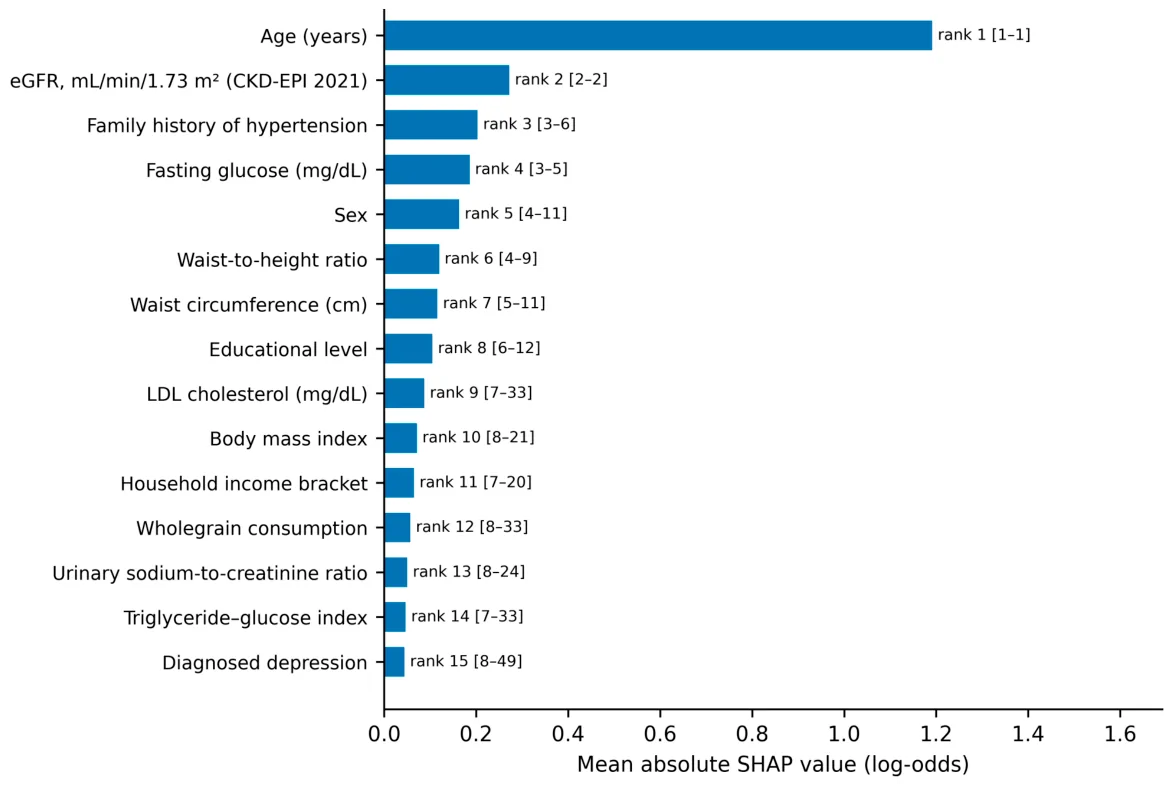
Mean absolute SHAP contribution of the fifteen highest-ranked model inputs to the output of the full XGBoost model, on the log-odds scale. Bars are annotated with the 2.5th-to-97.5th-percentile rank interval obtained from 25 refits on segment-level bootstrap resamples of the development data. Fifteen inputs are displayed for visual context; the twelve for which attribution is tabulated appear in Table 8. Only the first eight positions are reliably ordered; beyond that, rank intervals overlap so extensively that the displayed ordering should not be read as a ranking.

**Figure 6:**
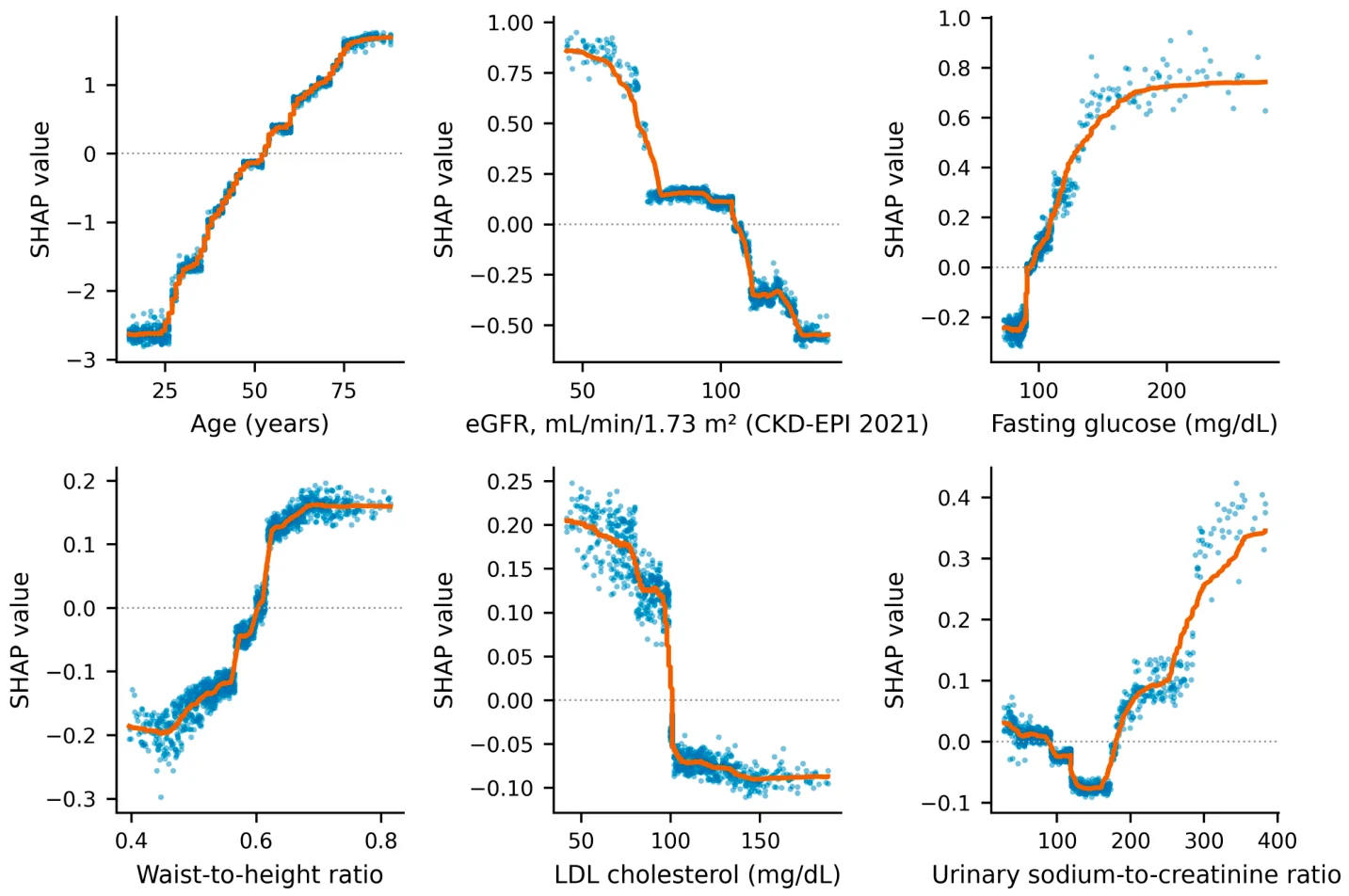
Dependence of individual SHAP contributions on input value for six model inputs, with a smoothed conditional mean overlaid; blue points are individual participant contributions, the orange line is the smoothed conditional mean and the dotted horizontal line marks zero contribution. The vertical axis is the SHAP contribution on the log-odds scale, where zero denotes no departure from the model base value of −0.4604; the horizontal axis is the input value, truncated at the 1st and 99th percentiles so that extreme values do not compress the informative range. Units are years for age, mL/min/1.73 m^2^ for estimated glomerular filtration rate (eGFR), mg/dL for glucose and LDL cholesterol, and dimensionless for the waist-to-height ratio. The urinary sodium-to-creatinine ratio is the native survey variable (Supplementary Table S1a, input 41), distinct from the derived sodium-to-potassium ratio (input 43) and is reported in the units supplied by the survey, which the official documentation does not specify; it is displayed for illustration and ranks thirteenth in Supplementary Table S5, below the eighth position beyond which we interpret no attribution. The step-like appearance reflects the finite number of split points used by gradient-boosted trees and is a property of the estimator, not of the data; the logistic specification, which imposes no such partition, achieves indistinguishable discrimination.

**Figure 7:**
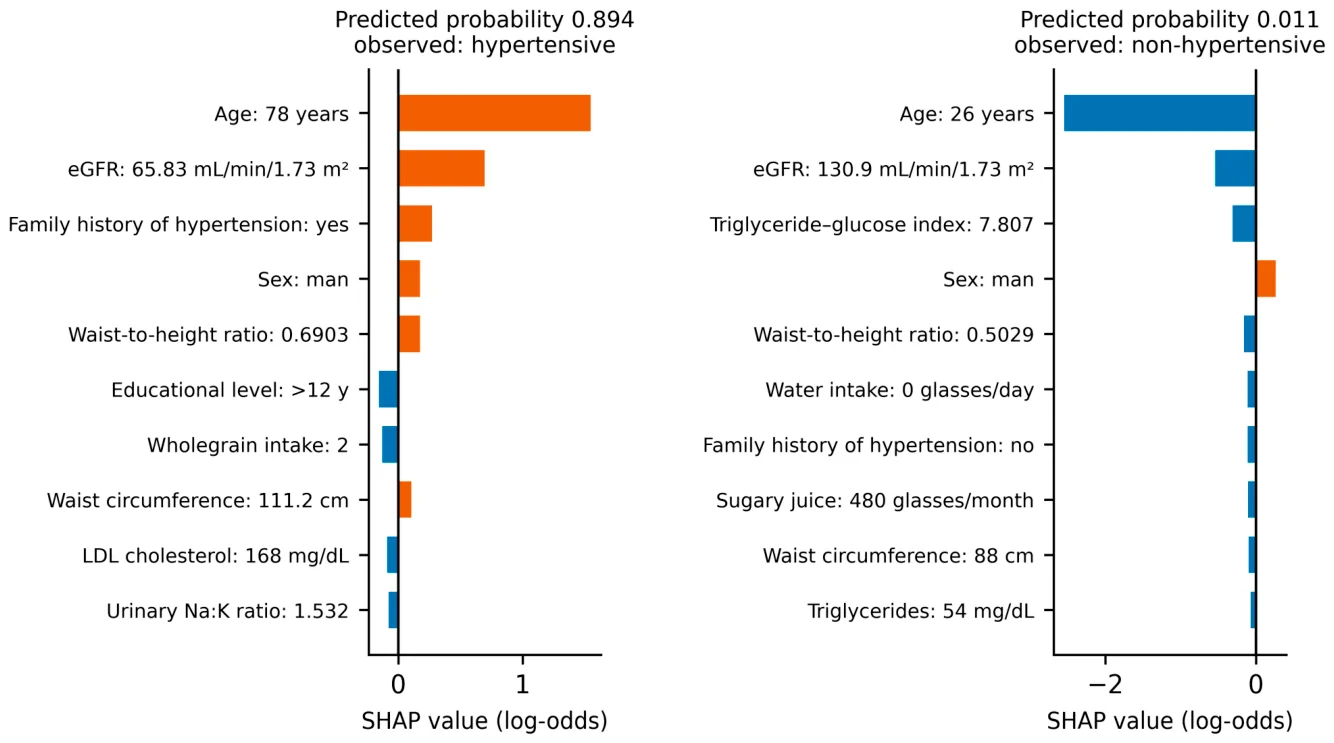
Contributions to the predicted risk of two representative participants, one at the 95th and one at the 5th percentile of predicted probability, showing the ten largest contributions with input values, the predicted probability, and the observed outcome. Positive contributions are shown in orange-red and negative in blue.

The distribution of predicted probabilities on the held-out partition, separated by observed outcome and annotated with each locked threshold, is shown in Supplementary Figure S2. A single SHAP computation on the held-out partition provides every main-effect attribution value reported below and in Figure 4, Figure 5, Figure 6 and Figure 7, so there is no possibility of numerical disagreement between the text, the tables and the figures. The pairwise interaction values reported in Section 3.10 were computed separately, on a random subsample, as specified in Section 2.13.

Age dominated, with a mean absolute SHAP value of 1.191 on the log-odds scale, 4.4 times that of the next-ranked input. Estimated glomerular filtration rate followed at 0.272, family history of hypertension at 0.203, fasting glucose at 0.186, and sex at 0.163 (Table 8, Figure 4 and Figure 5).

Participant-level attributions across the full held-out partition are displayed in Supplementary Figure S4 and the corresponding cumulative decision paths in Supplementary Figure S5.

Ten of the fifty-four inputs received a mean absolute SHAP value of exactly zero (financial stress, family history of diabetes, and eight of the eleven laboratory missingness indicators), meaning that the fitted XGBoost model never split on them. These ten are reported with the remainder in Supplementary Table S5, where they are tied at the bottom of the ordering. For the missingness indicators, this outcome is partly consistent with the structure of the input set rather than being an independent finding: two of these eight indicators, those for the total-to-HDL cholesterol ratio and for the sodium-to-potassium ratio, are deterministic functions of the indicators of their components and carry no information beyond them. The indicator of the third derived ratio, the triglyceride-glucose index, is not among the zero-valued inputs and ranks thirty-ninth with a mean absolute SHAP value of 0.0028, so redundancy of construction does not account for all eight zeros. The result is therefore compatible with, but does not by itself establish, the absence of survey-phase information in laboratory missingness.

Rank stability across 25 segment-level bootstrap refits was high only at the top of the ordering. Age and estimated glomerular filtration rate occupied ranks 1 and 2 in every resample; sex, nominally fifth, had a rank interval of 4 to 11 and educational level, nominally eighth, an interval of 6 to 12. Beyond approximately the eighth position, ranks became unstable; LDL cholesterol, nominally ninth, had a rank interval spanning 7 to 33. Only age and estimated glomerular filtration rate, which held ranks 1 and 2 in every resample, were fully stable; ranks 3 to 8 should be read together with their bootstrap intervals, and we interpret no attribution below the eighth position.

Direction of association was interpretable for 21 of the 54 inputs under the prespecified monotonicity rule. Of the remaining 33, 19 are binary inputs reported as a contrast between their two levels, 12 failed the monotonicity criterion and are marked non-monotonic, and 2 are nominal and are excluded by rule. Among those with a reportable direction, higher age, waist circumference, waist-to-height ratio, body mass index, and fasting glucose increased predicted risk, while higher estimated glomerular filtration rate, LDL cholesterol, and educational level decreased it. The inverse contribution of LDL cholesterol is addressed in the Section 4.

### 3.10. Interactions and Additivity

Exact pairwise SHAP interaction values were small relative to main effects. The strongest pairs were age with sex (mean absolute interaction 0.0244), age with waist circumference (0.0140), age with family history (0.0129), and estimated glomerular filtration rate with fasting glucose (0.0107). These magnitudes are one to two orders of magnitude below the corresponding main effects. The complete matrix is given as Supplementary Table S6 and displayed as Supplementary Figure S3.

A direct test confirms near-additivity. A depth-one XGBoost model, which cannot represent interactions by construction, achieved cross-validated AUC 0.8740 and held-out AUC 0.8888; the tuned interaction-enabled model achieved 0.8769 and 0.8898. The cross-validated difference of 0.0029 AUC (held-out difference 0.0010) provides an empirical estimate of the incremental discrimination associated with allowing interactions in this dataset. This estimate is bounded by the model class and the tuning space examined here and does not constitute a general decomposition of interaction effects.

### 3.11. Threshold Behaviour

**Figure 8:**
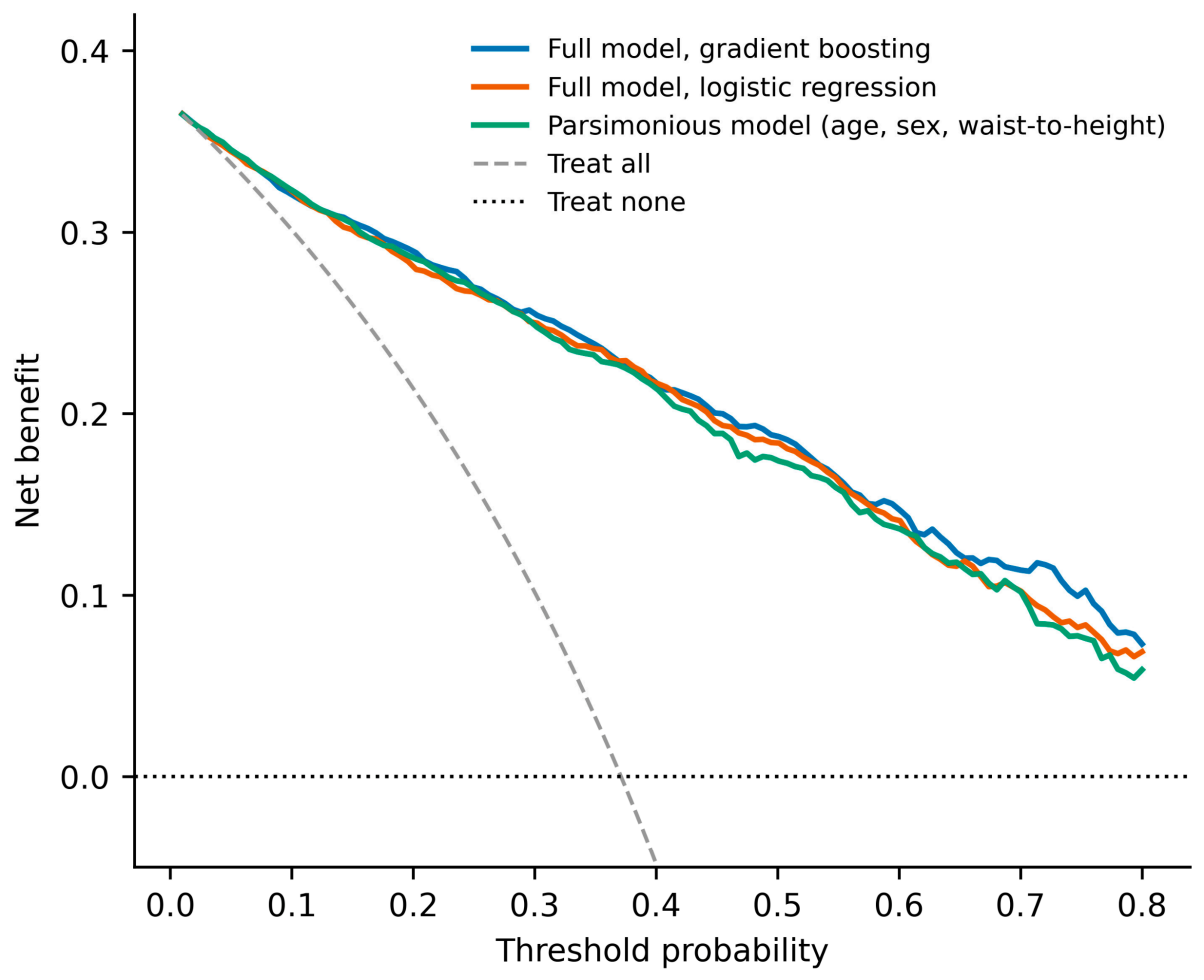
Net benefit across threshold probabilities for the three principal models on the held-out partition. This is a hypothetical mathematical illustration of threshold behaviour in this dataset and does not correspond to any real clinical decision, because blood pressure would necessarily be available wherever these models could be applied.

Figure 8 presents net benefit across threshold probabilities for the three principal models [43]. This is a hypothetical mathematical illustration of the threshold behaviour of the fitted models in this dataset. It does not correspond to any real clinical decision, because blood pressure—which defines the outcome—would necessarily be available in any setting where these models could be applied. We do not present it as evidence of clinical utility.

## 4. Discussion

In a nationally representative Chilean survey, a model containing age, sex, and waist-to-height ratio discriminated prevalent hypertension almost as well as fifty-four model inputs analysed by tuned machine learning pipelines. The difference was undetectable under a penalised logistic specification and approximately 0.008 AUC under XGBoost. Twelve algorithms tuned under an identical protocol spanned 0.012 in cross-validated AUC-ROC, a range comparable to the fold-to-fold variability of the individual algorithms rather than clearly exceeding it.

We state at the outset what this analysis does not establish. We did not prespecify an equivalence margin and did not test formal equivalence. A confidence interval that includes zero indicates insufficient evidence of a difference, not evidence of no difference. The intervals we report are compatible with the additional 51 inputs improving discrimination by as much as approximately 0.008 AUC under the logistic specification and 0.015 under the gradient-boosted one. Whether increments of that size matter are a clinical judgement rather than a statistical result; in our view, they would not alter any decision, but we present the range so that readers may judge for themselves.

Three features of the analysis give us some confidence that the finding is not an artefact. First, it survived every sensitivity analysis we could devise: design weighting, restriction to the laboratory subsample with and without phase weights, restriction to untreated participants with the outcome defined solely by measured blood pressure, restriction to participants aged 18 years and over, and the substitution of alternative single adiposity indices. Second, it did not depend on the specification: the parsimonious model was statistically indistinguishable from the full model under penalised logistic regression and differed by 0.0084 AUC under XGBoost, so the conclusion does not rest on the choice between a linear and a flexible estimator. Third, it is consistent with the structure of the fitted relationship, since exact pairwise interaction values were small relative to main effects and a depth-one model lost approximately 0.003 AUC, indicating that there is little non-additive structure available for a flexible algorithm to exploit.

Age dominated the attribution analysis by a factor of more than four over the next-ranked input, and removing it cost roughly ten times what the entire remaining panel contributes. It does not follow that the model is merely an age proxy: the age-free model still reached AUC 0.864, so the non-age inputs carry substantial joint discrimination, nor does the lower AUC within age bands support that reading. Conditioning on a dominant predictor removes case-mix heterogeneity and mechanically lowers AUC, and after accounting for uncertainty, no subgroup contrast in this study reached significance.

The inverse contribution of LDL cholesterol is a case in which the cross-sectional design is not merely a caveat but a plausible explanation. Lipid-lowering treatment was reported by 18.4% of hypertensive participants against 4.2% of non-hypertensive participants, and mean total cholesterol among treated participants was 179.8 mg/dL at a mean age of 63.2 years against 181.5 mg/dL at a mean age of 47.7 years among the untreated. Differential treatment is therefore a mechanism by which an inverse association could arise in these data; we did not test it formally, and the difference in mean total cholesterol between the two groups is itself small. No aetiological interpretation is warranted either way. The same logic applies with equal force to fasting glucose and to renal function, both of which may be consequences of long-standing elevated blood pressure or of antihypertensive therapy rather than antecedents of either. Throughout this manuscript, we refer to cross-sectional correlates and contributors to model output rather than to determinants.

That the fitted relationship is close to additive is consistent with the equivalence of the linear and flexible specifications. A model incapable of representing any interaction lost approximately 0.003 AUC relative to the tuned interaction-enabled model, and the largest exact interaction value we measured was more than an order of magnitude below the corresponding main effects. Where flexible algorithms confer little advantage, the explanation is often that there is little non-additive structure for them to exploit.

These findings sit alongside a systematic review of clinical prediction models that found no performance benefit of machine learning over logistic regression [44], and they carry a practical implication for settings where detection rather than treatment is the binding constraint. Age, sex and a waist measurement require a tape measure and two questions. If a screening instrument of that complexity performs comparably to one requiring venepuncture and a laboratory, the implication for resource allocation is direct—subject to the important qualification that nothing in this analysis speaks to incident hypertension, which is a different and more demanding prediction problem.

Assessing that equivalence on discrimination alone would have been inadequate, and doing so more broadly proved informative. Across AUC-ROC, AUC-PR, F1, balanced accuracy, and the Matthews correlation coefficient, the twelve tuned pipelines were closely comparable, with ranges, on the unrounded values, of 0.012, 0.026, 0.015, 0.012 and 0.020, respectively. Probability accuracy behaved differently: the Brier score ranged over 0.035 and the root mean squared error over 0.044, almost entirely because AdaBoost produced poorly scaled probabilities while ranking tenth out of twelve on discrimination. Upon excluding it, the Brier range narrows to 0.008. A comparison resting on AUC alone would have reported twelve broadly equivalent algorithms and missed this; we note this because the same blind spot is common in the applied machine learning literature, where discrimination is frequently the only axis reported.

### Limitations

Validation was internal. Held-out census segments came from the same survey, with the same sampling frame, the same field protocol, and the same measurement devices as the development data. This design excludes optimism from geographical clustering but demonstrates nothing about transportability. External validation would require an independent sample with an independent sampling frame and independent blood pressure ascertainment, and we did not perform it. The design of this study is cross-sectional. Predictors and outcome were measured at the same visit, so these models identify prevalent hypertension rather than predicting its onset, and none of the attributions support a causal reading. Model development was unweighted. Although design-weighted evaluation left discrimination essentially unchanged, calibration, predictive values, and threshold-dependent quantities depend on prevalence and are therefore anchored to the analytic sample rather than to the Chilean population.

The outcome incorporates current antihypertensive treatment, so 27.9% of the sample are classified as hypertensive partly on that basis, and half of those have controlled measured blood pressure. The untreated-only analysis addresses this directly and yields the same qualitative conclusions at lower absolute discrimination. Approximately one third of laboratory measurements were unavailable through planned subsampling. We addressed this with missingness indicators, with a complete-case analysis within the laboratory subsample and with laboratory-phase weights, and the conclusions were stable; nevertheless, attributions for individual laboratory inputs should be interpreted with corresponding caution.

Urinary sodium and potassium were estimated from spot samples rather than from 24 h collections, which are subject to substantial within-individual variability [45,46]. The adult diagnostic threshold of 140/90 mmHg was applied to the 214 participants aged 15 to 17, rather than the percentile-based paediatric criterion; the sensitivity analysis restricted to those aged 18 and over showed no material difference. Finally, we note that this manuscript supersedes an earlier analysis of the same data in which tables, figures and narrative were generated from different pipeline runs. The present results derive from a single locked execution, and none of the earlier values should be relied upon.

## 5. Conclusions

In this cross-sectional sample of the Chilean National Health Survey 2016–2017, age, sex, and waist-to-height ratio provided most of the discrimination achieved by fifty-four model inputs analysed with tuned machine learning pipelines. The remaining fifty-one inputs yielded no detectable increment under a linear specification and a small increment of approximately 0.008 AUC under a flexible one. We did not test formal equivalence and cannot exclude that those inputs carry real predictive information too small to detect at this sample size.

After comparable tuning, the better-performing conventional and ensemble methods performed similarly across seven metrics, and no pipeline ranked first on all of them. Probability accuracy was the one axis on which a candidate separated: AdaBoost produced a Brier score approximately 25% worse than the leading pipelines while ranking tenth of twelve on discrimination, a deficiency that reporting AUC alone would have concealed. We identify no best-performing algorithm, and formal equivalence was not tested for any of them.

Validation was internal, using held-out census segments from the same survey. No independent population was examined and transportability to other settings is undemonstrated. These models identify prevalent hypertension in participants whose blood pressure was measured at the same visit; they are not deployable prediction instruments, and none of the reported contributions support a causal interpretation.

## Figures and Tables

**Table 1:** Characteristics of the analytic sample (*n* = 5516) with hypertension frequency by category. Every categorical variable carries an explicit “not reported” row, so that categories sum to the stated total without exception. FONASA, Fondo Nacional de Salud, the Chilean public health insurance fund, whose beneficiaries are classified into income groups A to D; ISAPRE, Instituciones de Salud Previsional, the private insurers. FONASA covers 4621 participants (83.8%) and ISAPRE 499 (9.0%).

Characteristic	*n*	Hypertension, *n*	Hypertension, %
Total analytic sample	5516	2114	38.3
Sex—Men	2017	802	39.8
Sex—Women	3499	1312	37.5
Residence—Urban	4642	1713	36.9
Residence—Rural	874	401	45.9
Education—Low (<8 years)	1326	908	68.5
Education—Intermediate (8–12 years)	2947	948	32.2
Education—High (>12 years)	1196	237	19.8
Education—Not reported	47	21	44.7
Health insurance—FONASA group A	1339	532	39.7
Health insurance—FONASA group B	1484	722	48.7
Health insurance—FONASA group C	551	147	26.7
Health insurance—FONASA group D	532	168	31.6
Health insurance—FONASA, group not recorded	715	295	41.3
Health insurance—Armed forces	117	47	40.2
Health insurance—ISAPRE (private)	499	122	24.4
Health insurance—No insurance	209	56	26.8
Health insurance—Other system	6	3	50.0
Health insurance—Not reported	64	22	34.4
Age band: 15–44 years	2299	179	7.8
Age band: 45–64 years	1857	870	46.8
Age band: ≥65 years	1360	1065	78.3

**Table 6:** Held-out discrimination of penalised logistic models containing age, sex, and a single adiposity index, each fitted on the development partition. Intervals are 95% bootstrap intervals resampling census segments. The waist-to-height point estimate coincides with that of the parsimonious logistic model in Table 5 because the two models contain the same inputs; the intervals differ only because they were generated in separate bootstrap runs, and both are reported as computed rather than reconciled after the fact.

Adiposity Index	AUC	95% CI
Body mass index	0.876	0.856 to 0.895
Waist circumference	0.880	0.861 to 0.898
Waist-to-height ratio	0.881	0.862 to 0.898
Arm circumference	0.872	0.855 to 0.891

**Table 7:** Held-out discrimination of the full XGBoost model within strata, with 95% bootstrap intervals resampling census segments. Pairwise heterogeneity tests are reported in Supplementary Table S4. Two stratifications exclude participants whose stratifying variable was not recorded and therefore do not sum to the full held-out partition of 1412 participants and 524 events: educational level excludes 9 participants (3 events) and sums to 1403 participants and 521 events, and health insurance excludes 24 participants (7 events) and sums to 1388 participants and 517 events. Sex, age band, area of residence and obesity status each sum to the full partition. FONASA (public) comprises all beneficiaries of the Chilean public insurance fund, which is income groups A to D, together with those whose group was not recorded; non-FONASA comprises the armed forces scheme, ISAPRE private insurers, no insurance, and other systems. BMI is body mass index.

Stratum	*n*	Events	AUC (95% CI)
Sex—Women	893	332	0.895 (0.871, 0.915)
Sex—Men	519	192	0.882 (0.850, 0.909)
Age band, years—15–44	594	40	0.760 (0.685, 0.829)
Age band, years—45–64	480	218	0.742 (0.691, 0.782)
Age band, years—≥65	338	266	0.746 (0.685, 0.807)
Residence—Urban	1161	408	0.888 (0.866, 0.907)
Residence—Rural	251	116	0.893 (0.849, 0.928)
Educational level—High	304	61	0.903 (0.858, 0.948)
Educational level—Intermediate	760	239	0.868 (0.842, 0.891)
Educational level—Low	339	221	0.853 (0.811, 0.891)
Health insurance—FONASA (public)	1152	455	0.891 (0.872, 0.907)
Health insurance—Non-FONASA	236	62	0.867 (0.806, 0.921)
Obesity status—BMI ≥ 30	507	238	0.885 (0.853, 0.913)
Obesity status—BMI < 30	905	286	0.889 (0.865, 0.912)

**Table 8:** Contributions of the twelve highest-ranked model inputs to the output of the full XGBoost model on the held-out partition, with rank stability across 25 bootstrap refits resampling census segments in the development data. SHAP values are on the log-odds scale and carry no causal interpretation.

Rank	Model Input	Mean |SHAP|	Median Rank	Rank Interval (2.5th–97.5th)
1	Age	1.191	1	1 to 1
2	Estimated glomerular filtration rate	0.272	2	2 to 2
3	Family history of hypertension	0.203	4	3 to 6
4	Fasting glucose	0.186	3	3 to 5
5	Sex	0.163	5	4 to 11
6	Waist-to-height ratio	0.120	6	4 to 9
7	Waist circumference	0.116	7	5 to 11
8	Educational level	0.105	9	6 to 12
9	LDL cholesterol	0.087	12	7 to 33
10	Body mass index	0.071	12	8 to 21
11	Household income bracket	0.065	12	7 to 20
12	Wholegrain consumption	0.057	14	8 to 33

## Data Availability

The survey microdata are publicly available from the Chilean Ministry of Health. The complete analysis pipeline—comprising dataset construction, the locked partition, the algorithm comparison, hyperparameter optimisation, evaluation, all sensitivity analyses, the attribution analysis and every figure—is provided in the Supplementary Materials and will be deposited in a public repository with a citable identifier on acceptance, so that every value in this manuscript can be regenerated from the publicly available microdata.

## Supplementary Materials

The following supporting information can be downloaded at: https://www.mdpi.com/article/10.3390/diagnostics16172843/s1, Figure S1, participant flow and derivation of model inputs; Figure S2, distribution of predicted probabilities; Figure S3, exact pairwise SHAP interaction values for the twelve highest-ranked inputs; Figure S4, participant-level attribution heatmap with annotated clusters; Figure S5, full decision-path plot; Table S1a, the forty-three candidate predictors (model inputs 1 to 43), each with its source variable in the survey microdata, measurement scale, coding, completeness in the analytic sample and encoding; Table S1b, the eleven laboratory missingness indicators (model inputs 44 to 54), each with the laboratory variable whose observation it records and its completeness in the analytic sample; Note S1, Reproducibility, describing the fixed sequence of numbered analysis scripts executed under the single random seed, the programmatic assertion that excludes blood-pressure, diagnosis and treatment variables, and the software versions used; Table S2, balance between development and held-out partitions; Table S3, complete hyperparameter search spaces; Table S4, pairwise subgroup heterogeneity tests; Table S5, complete attribution values for all fifty-four model inputs, unweighted and design-weighted, with direction where interpretable; Table S6, exact pairwise SHAP interaction values; and the complete reproducible analysis pipeline.

## Abbreviations

AUC, area under the receiver operating characteristic curve; AUC-PR, area under the precision–recall curve; BMI, body mass index; CI, confidence interval; CKD-EPI, Chronic Kidney Disease Epidemiology Collaboration; ECE, expected calibration error; eGFR, estimated glomerular filtration rate; ENS, Encuesta Nacional de Salud (Chilean National Health Survey); FONASA, Fondo Nacional de Salud (Chilean public health insurance fund); HDL, high-density lipoprotein; ISAPRE, Instituciones de Salud Previsional (Chilean private health insurers); LDL, low-density lipoprotein; MCC, Matthews correlation coefficient; PROBAST, Prediction model Risk Of Bias Assessment Tool; RBF, radial basis function; RMSE, root mean squared error; SD, standard deviation; SHAP, SHapley Additive exPlanations; TRIPOD+AI, Transparent Reporting of a multivariable prediction model for Individual Prognosis Or Diagnosis, Artificial Intelligence extension; WHtR, waist-to-height ratio.
