# Supplementary material for: Age, Sex, and Waist-to-Height Ratio Approach the Discrimination of Fifty-Four Model Inputs for Prevalent Hypertension: An Explainable Machine Learning Analysis of the Chilean National Health Survey
Source: Diagnostics (Basel). 2026 Sep 4;16(17):2843. doi: 10.3390/diagnostics16172843 (PMC13565000; doi:10.3390/diagnostics16172843)
Supplement: Supplementary file 1 [file diagnostics-16-02843-s001.zip › diagnostics-4521810-supplementary.pdf]

diagnostics

MDPI

Supplementary Materials

Age, Sex, and Waist-to-Height Ratio Approach the Discrimination of Fifty-Four Model Inputs for Prevalent Hypertension: An Explainable Machine Learning Analysis of the Chilean National Health Survey

Manuscript diagnostics-4521810

All outputs in this document derive from the same single locked analysis pipeline as the main manuscript (random seed 42). Figures are 600 dpi RGB.

Candidate predictors and model inputs

The forty-three candidate predictors are listed individually with their source variable in the Chilean National Health Survey 2016–2017 microdata, measurement scale, coding, completeness in the analytic sample (n = 5516) and the encoding applied. Eleven binary indicators of laboratory missingness are added to these, giving fifty-four model inputs.

Table S1a. The forty-three candidate predictors. Under "Encoding", "Standardised / native" denotes standardisation for the scale-sensitive pipelines and native integer codes for the tree ensembles; "One-hot / native" denotes one-hot expansion for the scale-sensitive pipelines and native codes for the tree ensembles. One-hot expansion was applied to the three nominal inputs and to six of the seven binary inputs; physical activity, although binary, was passed through the standardisation path, and the two encodings are equivalent for a single binary column. All encoders were fitted inside each training fold.

#	Domain / model input	Source variable	Scale	Coding	Complete %	Encoding
	**Demography and socioeconomic position**					
1	age	Edad	Continuous	Years, 15–98	100.0	Standardised / native
2	sex	Sexo	Nominal	1 = men, 2 = women	100.0	One-hot / native
3	urban rural	Zona	Binary	1 = urban, 2 = rural	100.0	One-hot / native
4	education level	NEDU1_MINSAL_1	Ordinal	1 = low (<8 y), 2 = intermediate (8–12 y), 3 = high (>12 y)	99.1	Standardised / native
5	income bracket	as28	Ordinal	Eleven ascending monthly household income brackets, 1 = <CLP 78,000 to 11 = >CLP 1,573,000	85.2	Standardised / native
6	health insurance	as5_1	Nominal	1–5 FONASA (income groups A, B, C, D and group not recorded); 6 armed forces; 7 ISAPRE	98.8	One-hot / native

 https://doi.org/10.3390/xxxxx

#	Domain / model input	Source variable	Scale	Coding	Complete %	Encoding
				(private); 8 none; 9 other		
7	family history htn	af1a	Binary	1 = yes, 2 = no	94.9	One-hot / native
8	family history dm	af1b	Binary	1 = yes, 2 = no	96.1	One-hot / native
	**Adiposity**					
9	bmi	IMC	Continuous	kg/m²	99.3	Standardised / native
10	waist circumference	m4p3	Continuous	cm	99.3	Standardised / native
11	waist to height	m4p3 / m4p2	Continuous	Dimensionless ratio, derived	99.2	Standardised / native
12	arm circumference	m2p7	Continuous	cm	100.0	Standardised / native
	**Physical activity**					
13	physical activity gpaq	GPAQ	Binary	Derived from the official three-level Global Physical Activity Questionnaire (GPAQ) variable (1 low, 2 moderate, 3 high) as 0 = low, 1 = moderate or high	96.4	Standardised / native
	**Diet**					
14	fruit days	die6	Ordinal	Days per week, 0–7	100.0	Standardised / native
15	fruit portions	die7	Ordinal	Portions per day	93.2	Standardised / native
16	vegetable days	die8	Ordinal	Days per week, 0–7	100.0	Standardised / native
17	vegetable portions	die9	Ordinal	Portions per day	97.7	Standardised / native
18	fish frequency	die1a	Ordinal	Consumption frequency category	100.0	Standardised / native
19	dairy frequency	die2	Ordinal	Consumption frequency category	100.0	Standardised / native
20	dairy type	die3	Binary	1 = reduced-fat or skimmed, 2 = whole	92.0	One-hot / native
21	wholegrain frequency	die4	Ordinal	Consumption frequency category	100.0	Standardised / native
22	legume frequency	die5	Ordinal	Consumption frequency category	100.0	Standardised / native

#	Domain / model input	Source variable	Scale	Coding	Complete %	Encoding
23	water glasses	die11	Continuous	Glasses per day	99.9	Standardised / native
24	ssb monthly	die12_cantidad, die12_unidad, die12e	Continuous	Glasses per month, derived; unit code 4 = does not consume mapped to zero	100.0	Standardised / native
25	juice monthly	die13_cantidad, die13_unidad, die13e	Continuous	Glasses per month, derived; unit code 4 = does not consume mapped to zero	100.0	Standardised / native
26	cooking fat type	die14	Nominal	0 = none, 1 = vegetable oil, 2 = olive oil, 3 = lard, 4 = butter, 5 = margarine, 6 = other	100.0	One-hot / native
27	warning label use	die10_c	Ordinal	Front-of-pack warning labels: 1 = always to 5 = never (higher = less use)	100.0	Standardised / native
	**Tobacco**					
28	smoked 100 cigarettes	ta2	Binary	1 = yes, 2 = no	98.9	One-hot / native
29	current smoking	ta3	Ordinal	1 = daily, 2 = occasional, 3 = former, 4 = never	100.0	Standardised / native
	**Psychosocial**					
30	perceived stress	ps7_F1	Ordinal	1 = never to 4 = permanently stressed	100.0	Standardised / native
31	financial stress	ps8	Ordinal	1 = little or none, 2 = moderate, 3 = high	98.7	Standardised / native
32	diagnosed depression	sd28_F1	Binary	Physician-diagnosed depression, ever	99.1	One-hot / native
	**Laboratory**					
33	glucose	Glucosa	Continuous	mg/dL, fasting	92.4	Standardised / native
34	total cholesterol	Colesterol_Total	Continuous	mg/dL	67.1	Standardised / native
35	hdl cholesterol	Colesterol_HDL	Continuous	mg/dL	67.1	Standardised / native
36	ldl cholesterol	Colesterol_LDL_Calculado	Continuous	mg/dL, Friedewald-calculated (official label reads DIRECTO; verified calculated, see Methods)	66.6	Standardised / native

#	Domain / model input	Source variable	Scale	Coding	Complete %	Encoding
37	triglycerides	Trigliceridos	Continuous	mg/dL	67.1	Standardised / native
38	tyg index	Derived	Continuous	ln(triglycerides × glucose / 2)	64.9	Standardised / native
39	tc hdl ratio	Derived	Continuous	Total / HDL cholesterol	67.1	Standardised / native
40	egfr ckdepi	Creatinina_en_Sangre	Continuous	mL/min/1.73 m², CKD-EPI 2021 race-free	95.3	Standardised / native
41	sodium creatinine	Sodio_Creatinina	Continuous	Spot urine sodium-to-creatinine ratio, survey-supplied units	66.6	Standardised / native
42	potassium creatinine	Potasio_Creatinina	Continuous	Spot urine potassium-to-creatinine ratio, survey-supplied units	66.6	Standardised / native
43	sodium potassium ratio	Derived	Continuous	Urinary Na/creatinine ÷ K/creatinine	66.6	Standardised / native

Table S1b. The eleven binary laboratory missingness indicators. Each takes the value 1 when the corresponding laboratory measurement is absent. Together with Table S1a these constitute the fifty-four model inputs.

#	Missingness indicator	Derived from	Observed, %
44	miss_glucose	Glucosa	92.4
45	miss_total cholesterol	Colesterol_Total	67.1
46	miss_hdl cholesterol	Colesterol_HDL	67.1
47	miss_ldl cholesterol	Colesterol_LDL_Calculado	66.6
48	miss_triglycerides	Trigliceridos	67.1
49	miss_tyg index	Derived	64.9
50	miss_tc hdl ratio	Derived	67.1
51	miss_egfr ckdepi	Creatinina_en_Sangre	95.3
52	miss_sodium creatinine	Sodio_Creatinina	66.6
53	miss_potassium creatinine	Potasio_Creatinina	66.6
54	miss_sodium potassium ratio	Derived	66.6

Two further laboratory candidates were considered and not retained. Serum uric acid was not measured in this survey wave. Microalbuminuria was measured but is ascertained only within a subset of the laboratory phase; it was excluded because retaining it would have introduced a second missingness mechanism, structurally distinct from the planned laboratory subsampling carried by the eleven indicators. The exclusion does not

rest on the 65% completeness threshold, which is applied to the source laboratory measurements listed in Table S1a.

Variables excluded by design. The following were withheld from the predictor set and their absence is enforced by a programmatic assertion that halts the pipeline if any appears among the model inputs: all six blood-pressure readings (m2p8_1, m2p8_2, m2p9_1, m2p9_2, m2p10_1, m2p10_2 and any third-visit equivalents), self-reported hypertension diagnosis, and all antihypertensive treatment items (m2p1, m2p2, h2, h4, h5, h8).

https://doi.org/10.3390/xxxxx

Balance between development and held-out partitions

Table S2. Continuous variables are shown as mean (SD) and compared by Mann–Whitney test; categorical variables are shown as the percentage in the first level and compared by chi-squared test. Standardised differences below 0.10 are conventionally regarded as negligible. The single nominally significant difference, urban residence, is an expected consequence of randomising intact geographical clusters.

Variable	Type	Development (n = 4104)	Held-out (n = 1412)	Standardised difference	p
Age, years	Continuous	49.30 (19.24)	48.90 (19.34)	0.0209	0.479
Body mass index, kg/m²	Continuous	28.99 (5.61)	28.77 (5.49)	0.0382	0.198
Waist circumference, cm	Continuous	94.14 (14.11)	93.50 (13.71)	0.0461	0.248
Waist-to-height ratio	Continuous	0.59 (0.09)	0.59 (0.09)	0.0498	0.257
Fasting glucose, mg/dL	Continuous	99.86 (35.77)	100.34 (37.27)	−0.0129	0.788
Sex	Categorical	36.5% (level 1)	36.8% (level 1)	−0.0053	0.889
Urban residence	Categorical	84.8% (level 1)	82.2% (level 1)	0.0700	0.024
Educational level	Categorical	24.3% (level 1)	24.2% (level 1)	0.0026	0.966
Health insurance	Categorical	24.7% (level 1)	24.1% (level 1)	0.0133	0.637
Hypertension	Categorical	61.3% (level 0)	62.9% (level 0)	−0.0336	0.291
Census segments	Design	810	271	—	—

Complete hyperparameter search spaces

Every algorithm whose default cross-validated AUC fell within 0.02 of the leading default model proceeded to tuning. Randomised search was performed over the same segment-grouped cross-validation folds used for the default comparison. All distributions are given exactly as sampled; loguniform(a, b) denotes a log-uniform distribution on [a, b], uniform(a, w) a uniform distribution on [a, a + w], and randint(a, b) a discrete uniform distribution on the integers [a, b).

Table S3. Search spaces, configuration budgets and selected hyperparameters for the twelve tuned algorithms. Configuration budgets differ across algorithms according to computational cost and are reported rather than concealed. Distributions follow SciPy conventions: uniform(a, b) denotes a uniform distribution on the interval [a, a + b], and randint(a, b) denotes integers drawn uniformly from a to b - 1 inclusive. Thus subsample ~ uniform(0.6, 0.4) samples from [0.6, 1.0] and max_depth ~ randint(2, 7) samples from {2, 3, 4, 5, 6}. Performance of these tuned pipelines is reported once only, in main-text Table 3; it is deliberately not repeated here, so that a single value exists for each quantity across the whole submission.

Algorithm	Configurations	Search space	Selected values
Penalised logistic regression	30	C ~ loguniform(1e-3, 1e2); penalty ∈ {L1, L2}; solver = saga; max_iter = 5000	C=0.2157; penalty=l1
Linear discriminant analysis	30	shrinkage ~ uniform(0, 1); solver = lsqr	shrinkage=0.02058
Support vector machine (RBF)	15	C ~ loguniform(1e-2, 1e2); gamma ~ loguniform(1e-4, 1e0)	C=21.37; gamma=0.0007069
Gradient boosting (sklearn)	10	n_estimators ~ randint(100, 350); learning_rate ~ loguniform(0.01, 0.3); max_depth ~ randint(2, 7); subsample ~ uniform(0.6, 0.4); min_samples_leaf ~ randint(5, 60)	learning_rate=0.04345; max_depth=2; min_samples_leaf=31; n_estimators=158; subsample=0.7599
Extremely randomised trees	25	n_estimators ~ randint(200, 800); max_depth ~ randint(3, 20); min_samples_leaf ~ randint(1, 40); max_features ~ uniform(0.1, 0.9)	max_depth=9; max_features=0.4811; min_samples_leaf=1; n_estimators=671
Random forest	8	n_estimators ~ randint(150, 400); max_depth ~ randint(3, 20); min_samples_leaf ~ randint(1, 40); max_features ~ uniform(0.1, 0.9)	max_depth=9; max_features=0.8169; min_samples_leaf=15; n_estimators=256
Histogram gradient boosting	30	learning_rate ~ loguniform(0.01, 0.3); max_depth ~ randint(2, 8); max_leaf_nodes ~ randint(8, 64); min_samples_leaf ~ randint(5, 60); l2_regularization ~ loguniform(1e-3, 1e1)	l2_regularization=7.287; learning_rate=0.1564; max_depth=2; max_leaf_nodes=33; min_samples_leaf=57
LightGBM	30	n_estimators ~ randint(100, 800); learning_rate ~ loguniform(0.01, 0.3); max_depth ~ randint(2, 8); num_leaves ~ randint(8, 64); subsample ~ uniform(0.6, 0.4);	colsample_bytree=0.6988; learning_rate=0.05818; max_depth=2; n_estimators=572; num_leaves=57;

**Algorithm**	**Configurations**	**Search space**	**Selected values**
		colsample_bytree ~ uniform(0.5, 0.5); reg_lambda ~ loguniform(1e-3, 1e2)	reg_lambda=42.02; subsample=0.9509
XGBoost	30	n_estimators ~ randint(100, 800); learning_rate ~ loguniform(0.01, 0.3); max_depth ~ randint(2, 8); subsample ~ uniform(0.6, 0.4); colsample_bytree ~ uniform(0.5, 0.5); min_child_weight ~ randint(1, 20); reg_lambda ~ loguniform(1e-3, 1e2)	colsample_bytree=0.5257; learning_rate=0.0258; max_depth=2; min_child_weight=1; n_estimators=502; reg_lambda=2.532; subsample=0.8124
AdaBoost	25	n_estimators ~ randint(50, 500); learning_rate ~ loguniform(0.01, 1.5)	learning_rate=0.3474; n_estimators=199
Bagging	10	n_estimators ~ randint(50, 250); max_samples ~ uniform(0.5, 0.5); max_features ~ uniform(0.5, 0.5)	max_features=0.6873; max_samples=0.9754; n_estimators=156
CatBoost	15	iterations ~ randint(200, 700); learning_rate ~ loguniform(0.01, 0.3); depth ~ randint(3, 8); l2_leaf_reg ~ loguniform(1, 30)	depth=6; iterations=663; l2_leaf_reg=2.207; learning_rate=0.01361

Nested specifications were tuned independently over the same folds and the same search spaces reported above, without any reference to the held-out partition, using a reduced budget of 15 configurations under the XGBoost search space and 10 under the penalised logistic search space, against 30 for the corresponding models in main-text Table 3. This reduced budget is the reason the independently tuned full models in main-text Table 5 differ marginally from their counterparts in Table 4.

Subgroup heterogeneity in discrimination

Table S4. Pairwise differences in held-out discrimination between strata for the full XGBoost model. Differences and intervals were computed within 1000 bootstrap replicates resampling census segments, rather than the 2000 replicates used for the single-model intervals reported in the main text, because each replicate here requires a paired estimate in every stratum; the two-sided bootstrap p value is twice the smaller tail proportion. No contrast reached significance at the 5% level.

Stratifying variable	Contrast	Δ AUC	95% CI	Bootstrap p
Sex	Women vs Men	+0.0132	−0.0228 to +0.0507	0.476
Age band, years	15–44 vs 45–64	+0.0196	−0.0655 to +0.1026	0.618
Age band, years	15–44 vs ≥ 65	+0.0143	−0.0798 to +0.1057	0.726
Age band, years	45–64 vs ≥ 65	−0.0053	−0.0842 to +0.0725	0.918
Area of residence	Urban vs Rural	−0.0054	−0.0469 to +0.0428	0.782
Educational level	High vs Intermediate	+0.0361	−0.0156 to +0.0858	0.172
Educational level	High vs Low	+0.0492	−0.0113 to +0.1064	0.112
Educational level	Intermediate vs Low	+0.0131	−0.0338 to +0.0606	0.568
Health insurance	FONASA (public) vs Non-FONASA	+0.0237	−0.0330 to +0.0867	0.482
Obesity status	BMI < 30 vs BMI ≥ 30	+0.0033	−0.0331 to +0.0418	0.860

Complete attribution values for all fifty-four model inputs

Table S5. All fifty-four model inputs ranked by mean absolute SHAP value in the full XGBoost model on the held-out partition. The ten inputs whose mean absolute SHAP value is exactly zero are tied and share the mean of the positions they occupy, which is why the displayed ranks run to 44 and then jump to 49; no input occupies ranks 45 to 48. Unweighted and design-weighted columns are reported separately; the two orderings agree closely (Spearman rho = 0.999 between the unweighted and weighted magnitudes). Sex-stratified columns are computed from the same single fitted model and the same single expected value (base value −0.4604 on the log-odds scale), so the two strata are directly comparable. The rank interval is the 2.5th to 97.5th percentile across 25 bootstrap refits resampling census segments in the development data. Direction is reported only where interpretable under the prespecified monotonicity rule stated in Methods section 2.13: nominal inputs are excluded, binary inputs are described as a level contrast, and ordinal or continuous inputs receive a direction only when the sign of the Spearman coefficient agrees with the sign of the change in mean SHAP value across at least three of the four adjacent quintile transitions. Use of front-of-pack warning labels is reverse-coded in the survey (1 = always to 5 = never), so a positive direction for that input denotes less frequent label use. Ten inputs received a mean absolute SHAP value of exactly zero, meaning the fitted model never split on them; these are tied at the bottom of the ordering and are assigned the mid-rank of that tie (49 of 54), which is also why their bootstrap rank intervals do not extend to 54. Eight of the ten are laboratory missingness indicators, two of which (the total-to-HDL cholesterol ratio and the sodium-to-potassium ratio) are deterministic functions of other indicators in the same set; the indicator of the triglyceride-glucose index, the third derived ratio, is not among the zeros and ranks thirty-ninth. This redundancy is a property of the input construction and is discussed in Section 3.9 of the main text. SHAP values are on the log-odds scale and carry no causal interpretation.

Rank	Model input	Mean |SHAP|	Weighted	Wtd. rank	Rank interval	Men	Women	Direction
1	age	1.1913	1.2749	1	1–1	1.2705	1.1452	Higher increases risk
2	egfr ckdepi	0.2721	0.2771	2	2–2	0.2610	0.2786	Higher decreases risk
3	family history htn	0.2033	0.2023	3	3–6	0.1998	0.2052	Level contrast only
4	glucose	0.1858	0.1785	5	3–5	0.1731	0.1931	Higher increases risk
5	sex	0.1630	0.1822	4	4–11	0.2159	0.1322	Level contrast only
6	waist to height	0.1197	0.1163	7	4–9	0.1175	0.1209	Higher increases risk
7	waist circumference	0.1156	0.1222	6	5–11	0.1126	0.1173	Higher increases risk
8	education level	0.1050	0.1007	8	6–12	0.0989	0.1086	Higher decreases risk
9	ldl cholesterol	0.0875	0.0869	9	7–33	0.0869	0.0878	Higher decreases risk
10	bmi	0.0712	0.0718	10	8–21	0.0690	0.0725	Higher increases risk

Rank	Model input	Mean |SHAP|	Weighted	Wtd. rank	Rank interval	Men	Women	Direction
11	income bracket	0.0647	0.0584	12	7–20	0.0637	0.0653	Non-monotonic
12	wholegrain frequency	0.0568	0.0598	11	8–33	0.0555	0.0575	Higher increases risk
13	sodium creatinine	0.0505	0.0463	14	8–24	0.0473	0.0523	Non-monotonic
14	tyg index	0.0468	0.0471	13	7–33	0.0451	0.0478	Non-monotonic
15	diagnosed depression	0.0444	0.0438	15	8–49	0.0324	0.0514	Level contrast only
16	health insurance	0.0364	0.0363	16	9–29	0.0384	0.0353	Not applicable (nominal)
17	triglycerides	0.0302	0.0309	17	13–31	0.0331	0.0286	Non-monotonic
18	sodium potassium ratio	0.0261	0.0261	19	10–34	0.0255	0.0264	Higher increases risk
19	ssb monthly	0.0260	0.0265	18	13–38	0.0291	0.0242	Higher decreases risk
20	current smoking	0.0213	0.0224	20	14–43	0.0227	0.0205	Higher increases risk
21	fruit portions	0.0195	0.0190	21	17–43	0.0206	0.0188	Non-monotonic
22	miss glucose	0.0177	0.0146	24	18–49	0.0168	0.0181	Level contrast only
23	water glasses	0.0174	0.0173	22	15–36	0.0169	0.0177	Non-monotonic
24	arm circumference	0.0152	0.0151	23	14–34	0.0144	0.0157	Non-monotonic
25	physical activity gpaq	0.0140	0.0136	25	13–50	0.0152	0.0134	Level contrast only
26	warning label use	0.0124	0.0127	26	16–45	0.0123	0.0125	Higher decreases risk
27	potassium creatinine	0.0117	0.0120	27	17–39	0.0109	0.0122	Higher decreases risk
28	juice monthly	0.0115	0.0104	28	13–36	0.0119	0.0113	Higher decreases risk
29	hdl cholesterol	0.0105	0.0093	31	18–35	0.0081	0.0119	Non-monotonic
30	vegetable days	0.0101	0.0099	29	9–43	0.0106	0.0098	Higher decreases risk
31	tc hdl ratio	0.0093	0.0097	30	11–37	0.0102	0.0088	Higher decreases risk
32	total cholesterol	0.0084	0.0086	32	9–35	0.0086	0.0083	Higher decreases risk
33	legume frequency	0.0071	0.0068	33	14–42	0.0068	0.0072	Higher decreases risk
34	vegetable portions	0.0065	0.0067	34	16–43	0.0053	0.0071	Non-monotonic
35	fish frequency	0.0046	0.0044	37	28–43	0.0047	0.0046	Non-monotonic
36	fruit days	0.0042	0.0046	35	22–42	0.0051	0.0037	Higher decreases risk

Rank	Model input	Mean |SHAP|	Weighted	Wtd. rank	Rank interval	Men	Women	Direction
37	dairy type	0.0040	0.0036	38	16–49	0.0037	0.0043	Level contrast only
38	dairy frequency	0.0038	0.0044	36	15–40	0.0044	0.0035	Non-monotonic
39	miss tyg index	0.0028	0.0028	39	30–51	0.0027	0.0028	Level contrast only
40	cooking fat type	0.0028	0.0019	42	24–50	0.0027	0.0028	Not applicable (nominal)
41	perceived stress	0.0023	0.0025	40	16–50	0.0020	0.0024	Higher increases risk
42	miss potassium creatinine	0.0021	0.0021	41	37–51	0.0018	0.0022	Level contrast only
43	smoked 100 cigarettes	0.0007	0.0008	43	27–51	0.0008	0.0007	Level contrast only
44	urban rural	0.0001	0.0001	44	27–51	0.0001	0.0002	Level contrast only
49	financial stress	0.0000	0.0000	49	20–50	0.0000	0.0000	Non-monotonic
49	family history dm	0.0000	0.0000	49	21–49	0.0000	0.0000	Level contrast only
49	miss total cholesterol	0.0000	0.0000	49	36–51	0.0000	0.0000	Level contrast only
49	miss hdl cholesterol	0.0000	0.0000	49	40–51	0.0000	0.0000	Level contrast only
49	miss triglycerides	0.0000	0.0000	49	41–51	0.0000	0.0000	Level contrast only
49	miss ldl cholesterol	0.0000	0.0000	49	45–51	0.0000	0.0000	Level contrast only
49	miss egfr ckdepi	0.0000	0.0000	49	42–51	0.0000	0.0000	Level contrast only
49	miss tc hdl ratio	0.0000	0.0000	49	48–51	0.0000	0.0000	Level contrast only
49	miss sodium creatinine	0.0000	0.0000	49	36–51	0.0000	0.0000	Level contrast only
49	miss sodium potassium ratio	0.0000	0.0000	49	39–51	0.0000	0.0000	Level contrast only

Exact pairwise SHAP interaction values

Interaction values were computed by TreeExplainer on a random sample of 400 held-out participants for the twelve highest-ranked model inputs. Entries are mean absolute interaction values on the log-odds scale; the matrix is symmetric and diagonal elements (main effects) are set to zero so that the table reflects interaction only.

Table S6. Mean absolute SHAP interaction values among the twelve highest-ranked model inputs. The largest value, age with sex (0.0244), is approximately fifty times smaller than the mean absolute main effect of age (1.191) and approximately seven times smaller than that of sex (0.163). Across the twelve inputs examined, every pairwise interaction is smaller than both of its constituent main effects, and the largest interactions are one to two orders of magnitude below the dominant main effects; this is the basis for the statement in the main text that the fitted relationship is close to additive. Values were computed on a random sample of 400 held-out participants, as specified in Methods Section 2.13. Abbreviations: eGFR, estimated glomerular filtration rate; Fam. hist., family history of hypertension; WHtR, waist-to-height ratio; Waist, waist circumference; LDL-C, LDL cholesterol; BMI, body mass index; Income, household income bracket; Wholegrain, wholegrain consumption.

	**Age**	**eGFR**	**Fam. hist.**	**Glucose**	**Sex**	**WHtR**	**Waist**	**Education**	**LDL-C**	**BMI**	**Income**	**Wholegrain**
Age	0.0000	0.0032	0.0129	0.0032	0.0244	0.0035	0.0140	0.0000	0.0068	0.0051	0.0021	0.0011
eGFR	0.0032	0.0000	0.0004	0.0107	0.0051	0.0074	0.0024	0.0064	0.0005	0.0025	0.0015	0.0009
Fam. hist.	0.0129	0.0004	0.0000	0.0066	0.0000	0.0047	0.0014	0.0067	0.0036	0.0000	0.0000	0.0000
Glucose	0.0032	0.0107	0.0066	0.0000	0.0008	0.0085	0.0015	0.0064	0.0015	0.0048	0.0025	0.0006
Sex	0.0244	0.0051	0.0000	0.0008	0.0000	0.0006	0.0000	0.0048	0.0000	0.0019	0.0062	0.0006
WHtR	0.0035	0.0074	0.0047	0.0085	0.0006	0.0000	0.0001	0.0015	0.0017	0.0000	0.0015	0.0035
Waist	0.0140	0.0024	0.0014	0.0015	0.0000	0.0001	0.0000	0.0014	0.0023	0.0000	0.0022	0.0017
Education	0.0000	0.0064	0.0067	0.0064	0.0048	0.0015	0.0014	0.0000	0.0010	0.0000	0.0000	0.0000
LDL-C	0.0068	0.0005	0.0036	0.0015	0.0000	0.0017	0.0023	0.0010	0.0000	0.0000	0.0000	0.0000
BMI	0.0051	0.0025	0.0000	0.0048	0.0019	0.0000	0.0000	0.0000	0.0000	0.0000	0.0008	0.0025
Income	0.0021	0.0015	0.0000	0.0025	0.0062	0.0015	0.0022	0.0000	0.0000	0.0008	0.0000	0.0000
Wholegrain	0.0011	0.0009	0.0000	0.0006	0.0006	0.0035	0.0017	0.0000	0.0000	0.0025	0.0000	0.0000

Supplementary Figures

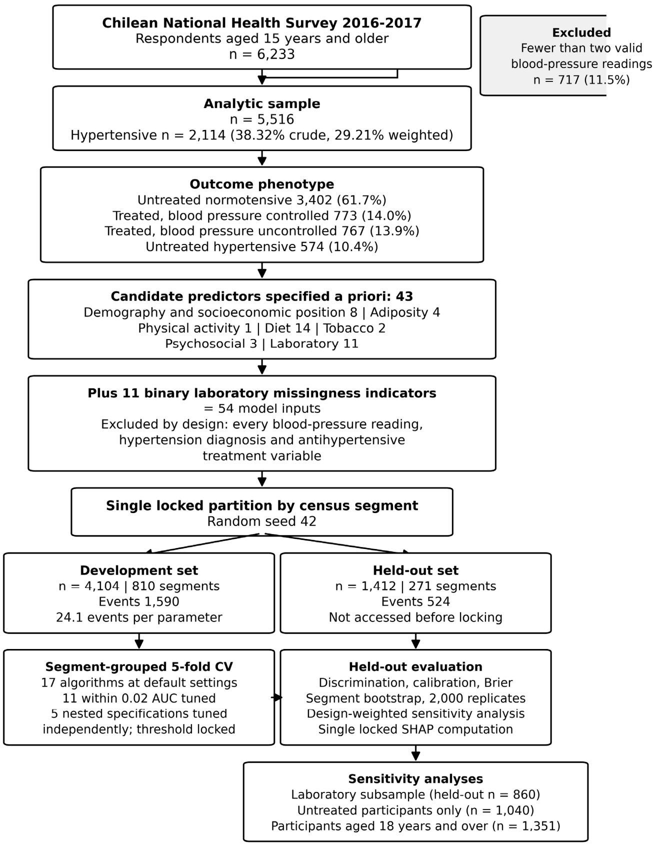

Figure S1. Participant flow and derivation of model inputs, from the 6233 survey respondents to the 5516-participant analytic sample, and from the forty-three candidate predictors to the fifty-four model inputs. The exclusion of all blood-pressure, diagnosis and treatment variables is enforced programmatically.

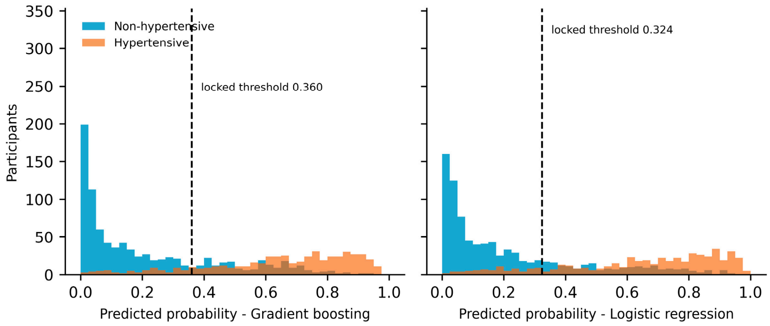

Figure S2. Distribution of predicted probabilities on the held-out partition for the two principal full models, separated by observed outcome. The dashed line marks the operating threshold derived from development out-of-fold predictions and locked before evaluation.

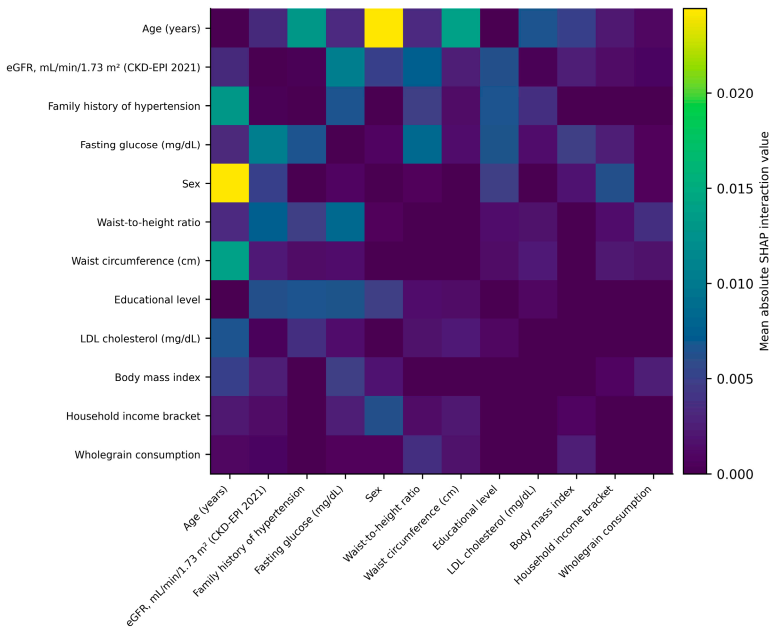

Figure S3. Exact pairwise SHAP interaction values among the twelve highest-ranked model inputs. Because exact interaction computation scales quadratically in the number of inputs, these values were computed by TreeExplainer on a random sample of 400 held-out participants drawn under the same seed, and are the only attribution values in this submission not derived from the single full-partition computation described in Methods Section 2.13. The colour scale encodes the mean absolute interaction on the log-odds scale; diagonal elements are set to zero so that the scale reflects interaction rather than main effects. The largest value, age with sex, is approximately fifty times smaller than the main effect of age and approximately seven times smaller than that of sex.

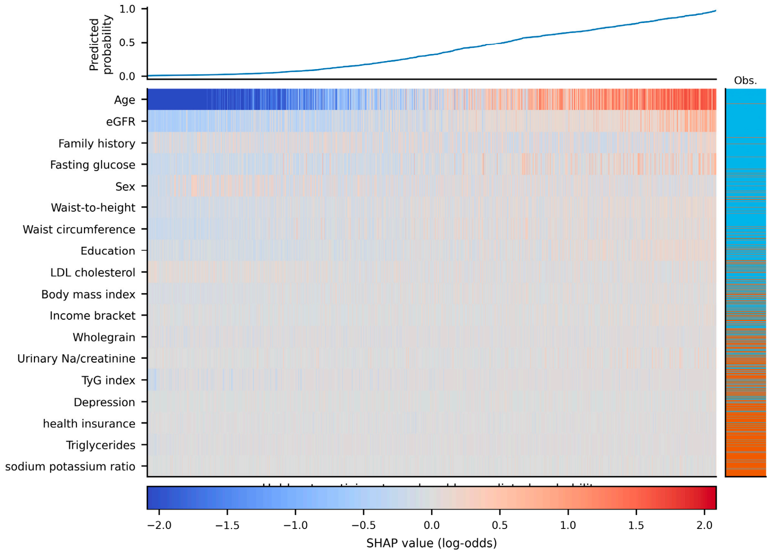

Figure S4. Participant-level attribution across all 1412 held-out participants, displaying the eighteen highest-ranked inputs for visual context against the twelve tabulated in main-text Table 8, ordered along the horizontal axis by predicted probability. The upper trace shows the predicted probability in that same order; the narrow bar to the right of the heatmap encodes the observed outcome (vermillion, hypertensive; light blue, non-hypertensive). The dominant vertical structure is the age band, which changes sign monotonically across the ordering; no other input shows comparable structure.

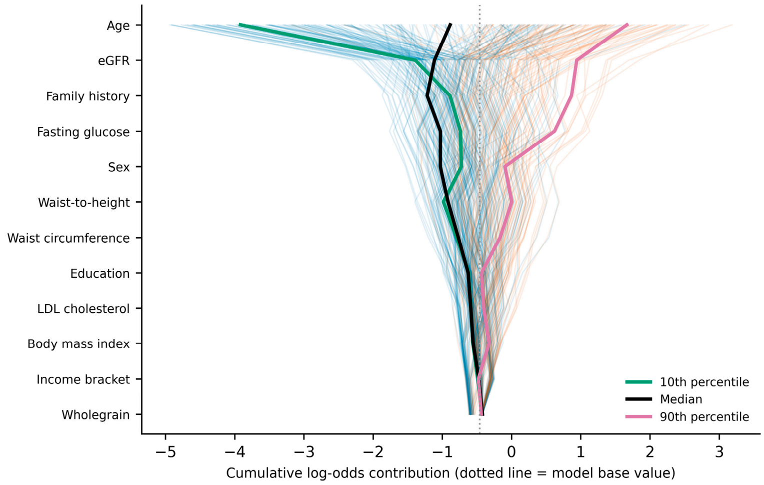

Figure S5. Cumulative decision paths for 300 randomly selected held-out participants across the twelve highest-ranked inputs, coloured by observed outcome (vermillion, hypertensive; blue, non-hypertensive). Three reference paths at the 10th, 50th and 90th percentiles of predicted probability are overlaid. The dotted vertical line marks the model base value. This figure is presented for completeness; the overplotting it exhibits is the reason the representative waterfall plots of main-text Figure 7 are preferred.

Supplementary Note S1. Reproducibility

The analysis is a fixed sequence of numbered scripts executed under a single random seed (42). Script 01 constructs the analytic dataset from the survey file and asserts that no blood-pressure, diagnosis or treatment variable appears among the model inputs. Script 02 draws the single development/held-out partition by census segment and performs the seventeen-algorithm comparison. Script 03 performs hyperparameter optimisation. Scripts 04a to 04c perform the held-out evaluation, the independently tuned nested specifications and all sensitivity analyses. Script 05 performs the single SHAP computation whose output is the sole source of every attribution value in the manuscript. Scripts 06 and 08 generate all figures, and script 07 performs the sample-size verification, the partition balance assessment, the additive-versus-interaction comparison and the monotonicity screening.

No value in the manuscript or in this document is transcribed by hand; all tables are written directly from the arrays produced by these scripts. The complete pipeline accompanies this submission and will be deposited in a public repository with a citable identifier on acceptance. The survey microdata are publicly available from the Chilean Ministry of Health, so every reported value can be regenerated independently.

Software versions: Python 3.12; scikit-learn 1.8.0; XGBoost 3.4.1; LightGBM 4.7.0; CatBoost 1.2.10; shap 0.52.0; statsmodels 0.14.6; pandas 3.0.2; NumPy 2.4.4; SciPy 1.17.1; Matplotlib 3.10.9.
